# Disabled-2 is a negative immune regulator of lipopolysaccharide-stimulated Toll-like receptor 4 internalization and signaling

**DOI:** 10.1038/srep35343

**Published:** 2016-10-17

**Authors:** Wei-Shan Hung, Pin Ling, Ju-Chien Cheng, Shy-Shin Chang, Ching-Ping Tseng

**Affiliations:** 1 Graduate Institute of Biomedical Sciences, College of Medicine, Chang Gung University, Kwei-Shan, Taoyuan 333, Taiwan, Republic of China; 2 Institute of Basic Medical Sciences, College of Medicine, National Cheng Kung University, Tainan 701, Taiwan, Republic of China; 3 Department of Microbiology and Immunology, College of Medicine, National Cheng Kung University, Tainan 701, Taiwan, Republic of China; 4 Department of Medical Laboratory Science and Biotechnology, China Medical University, Taichung 404, Taiwan, Republic of China; 5 Department of Family Medicine, Chang Gung Memorial Hospital, Kweishan, Taoyuan 333, Taiwan, Republic of China; 6 Department of Medical Biotechnology and Laboratory Science, College of Medicine, Chang Gung University, Kwei-Shan, Taoyuan 333, Taiwan, Republic of China; 7 Molecular Medicine Research Center, Chang Gung University, Kwei-Shan, Taoyuan 333, Taiwan, Republic of China; 8 Department of Laboratory Medicine, Chang Gung Memorial Hospital, Kwei-Shan, Taoyuan 333, Taiwan, Republic of China

## Abstract

Toll-like receptor 4 (TLR4) plays a pivotal role in the host response to lipopolysaccharide (LPS), a major cell wall component of Gram-negative bacteria. Here, we elucidated whether the endocytic adaptor protein Disabled-2 (Dab2), which is abundantly expressed in macrophages, plays a role in LPS-stimulated TLR4 signaling and trafficking. Molecular analysis and transcriptome profiling of RAW264.7 macrophage-like cells expressing short-hairpin RNA of Dab2 revealed that Dab2 regulated the TLR4/TRIF pathway upon LPS stimulation. Knockdown of Dab2 augmented TRIF-dependent interferon regulatory factor 3 activation and the expression of subsets of inflammatory cytokines and interferon-inducible genes. Dab2 acted as a clathrin sponge and sequestered clathrin from TLR4 in the resting stage of macrophages. Upon LPS stimulation, clathrin was released from Dab2 to facilitate endocytosis of TLR4 for triggering the TRIF-mediated pathway. Dab2 functions as a negative immune regulator of TLR4 endocytosis and signaling, supporting a novel role for a Dab2-associated regulatory circuit in controlling the inflammatory response of macrophages to endotoxin.

Innate immune responses are the first line of defense in fighting against invasion of pathogenic microbes. Binding of the pathogen-associated molecular patterns (PAMPs) that are expressed on infectious microorganisms to the pattern-recognition receptors in macrophages is crucial for the activation of macrophages and the production of cytokines and chemokines necessary for microbial clearance and the development of effective immunity^1^. Excessive generation of inflammatory mediators contributes to the pathogenesis of septic shock and autoimmune diseases, such as rheumatoid arthritis, inflammatory bowel disease, and multiple sclerosis^2^,^3^,^4^. Tight regulation of inflammatory responses is essential for the appropriate action of the innate immune system.

Toll-like receptors (TLRs) are the primary pattern-recognition receptors that act as the sensors of invading pathogens in macrophages and are pivotal to both innate and adaptive immunity^5^,^6^. Ligand binding results in the engagement of TLRs and activates multiple signaling cascades that ultimately cause the induction of genes involved in innate immune responses. At the initial stage of TLR signaling, specific combinations of the Toll/interleukin-1 receptor (TIR) domain-containing adapter proteins, such as myeloid differentiation factor 88 (MyD88), TIR-domain-containing adaptor protein-inducing interferon-β (TRIF), TIR-associated protein (TIRAP), and TRIF-related adaptor molecule (TRAM), are recruited to associate with individual TLRs. MyD88 is recruited to all TLRs, with the exception of TLR3. MyD88 interacts with interleukin-1 receptor-associated kinase (IRAK) complex and tumor necrosis factor receptor-associated factor 6 (TRAF6), resulting in activation of the canonical I kappa B kinase (IKK), nuclear factor-κB (NF-κB) and the mitogen-activated protein kinase (MAPK) cascade that is responsible for the formation of AP-1 transcription factor complex^7^. Alternatively, specific TLRs, including TLR3 and TLR4, recruit TRIF and, via TRAF3, induce the expression of cytokine genes through the activation of noncanonical IKKs and NF-κB, whereas the induction of type I interferon (IFN) and RANTES occurs through the phosphorylation and activation of IFN regulatory factor 3 (IRF3)^8^,^9^. Among the TLRs, TLR4 together with myeloid differentiation factor 2 (MD2) recognizes lipopolysaccharide (LPS), a principal membrane component of Gram-negative bacteria. Through the sorting adaptor of TIRAP, TLR4 recruits MyD88 and activates the MyD88-dependent pathway in response to LPS binding. After endocytosis of the TLR4/MD2 complex and through the sorting adaptor of TRAM to recruit TRIF, TLR4 signaling transits sequentially into a TRIF-dependent pathway that activates IRF3 and the generation of type I IFN and RANTES^9^,^10^,^11^.

Trafficking of TLR4 from the cell surface to the endosome/lysosome and from the endoplasmic reticulum (ER) to the cell surface are both important in the regulation of TLR4 signaling. In response to ligand binding, internalization of the surface TLR4/MD2 receptor complex into lysosomes not only activates TRIF-dependent signaling but also leads to the degradation of TLR4 and the termination of the LPS response. Clathrin-coated vesicles, dynamin, CD14 and Rab11a GTPase play a role in the internalization of the surface TLR4^12^,^13^,^14^,^15^. Maintenance of an optimal level of the surface TLR4 via continuous replenishment of TLR4 from intracellular compartments such as the Golgi apparatus and endosomes is also crucial for macrophage activation upon infection by Gram-negative bacteria. Both chaperones gp96 and PRAT4A are key players in TLR4 trafficking from the ER to the cell surface^16^,^17^. The small GTPase Rab10 further refines TLR4 signaling by regulating the trafficking rate of TLR4 moving to the plasma membrane^18^.

Disabled-2 (Dab2) is an endocytic adaptor protein involved in the regulation of receptor trafficking of the low-density lipoprotein receptor (LDLR)^19^,^20^, the apolipoprotein E receptor 2 (ApoER2)^21^, megalin^22^,^23^,^24^, integrin β1 and αIIbβ3^25^,^26^,^27^,^28^, the type II transforming growth factor-β receptor^29^, the cystic fibrosis transmembrane conductance regulator (CFTR)^30^ and the MHC I & II molecules^31^. Dab2 facilitates the assembly of the cargo transmembrane receptors to the clathrin-coated pits and regulates receptor internalization by engaging the N-terminal phosphotyrosine binding domain of Dab2 with the NPXY motif in the cargo transmembrane receptors^28^,^32^. Dab2 is widely distributed in various immune cell types including macrophages, T cells and dendritic cells, where they have different functions. Dab2 mediates the signal transduction of colony stimulating factor 1 (CSF-1) in macrophages^33^,^34^, functions as a negative regulator in adaptive immunity and is a FOXP3 target gene that is required for a subset of T_reg_ cell functions^35^. Vaccination with Dab2-deficient dendritic cells inhibits tumor growth by negatively regulating immunogenicity in dendritic cells^31^. Regardless of these studies, the role and the underlying mechanisms of Dab2 in the innate immune response and the trafficking of TLR4 has yet to be established.

This study revealed that Dab2 acts as a negative regulator in TLR4 signaling by restricting the clathrin interaction with TLR4, indicating a new role of Dab2 in the inflammatory response of macrophages.

## Materials and Methods

### Materials and reagents

Escherichia coli LPS (serotype O111:B4) was obtained from Sigma (St. Louis, MO). Murine macrophage colony-stimulating factor (M-CSF) was purchased from PeproTech (Rehovot, Israel). R-phycoerythrin (PE)-Texas Red-conjugated rat anti-IgG2aκ antibody and anti-F4/80 (BM8) antibody were purchased from Molecular Probe (Waltham, MA). Anti-Dab2 (p96) antibody was purchased from BD Biosciences (San Jose, CA). The anti*-*phospho-p38 (Thr180/Tyr182), anti-phospho-ERK1/2 (Thr202/Tyr204), anti-phospho-SAPK/JNK (Thr183/Tyr185), anti-IRAK1, anti-phospho-IRF3 (S396), anti-p65 and anti-TLR4 (#14358) antibodies were purchased from Cell Signaling Technology (Beverly, MA). The anti-IκBα and anti-Dab2 (H-110) antibodies were purchased from Santa Cruz Biotechnology (Dallas, Texas). The anti-IRF-3 antibody was purchased from Epitomics (Burlingame, CA). The anti-β-actin antibody was purchased from Novus Biologicals (Littleton, CO). The anti-clathrin heavy chain (CHC) antibody was purchased from Abcam (Cambridge, UK). The anti-mouse CD16/32 (FcγRIII/II) and biotin-conjugated rat anti-TLR4/MD2 (MTS510) antibodies, the rat IgG2a isotype control, and the PE-conjugated streptavidin were purchased from eBioscience (San Diego, CA). The biotin-conjugated rat anti-TLR4 antibody (Sa15–21) was a gift from Dr. Kensuke Miyake (University of Tokyo). The PE-conjugated anti-mouse CD64 (FcγRI) antibody was purchased from BioLegend (San Diego, CA). The horseradish peroxidase (HRP)-conjugated anti-mouse and anti-rabbit secondary antibodies and the chemiluminescent HRP substrate were purchased from Millipore (Darmstadt, Germany). The shRNA plasmids were obtained from the National RNAi Core Facility located at the Institute of Molecular Biology/Genomic Research Center, Academia Sinica, and supported by the National Core Facility Program for Biotechnology Grants of NSC (NSC 100-2319-B-001-002).

### Cell Culture

The RAW 264.7 murine macrophage cell line was maintained in macrophage growth medium (Dulbecco’s modified Eagle medium (DMEM) supplemented with 10% heat-inactivated low-endotoxin fetal bovine serum, 4 mM L-glutamine, 100 U/ml penicillin, and 100 μg/ml streptomycin). Murine bone marrow-derived macrophages (BMMs) were obtained as described previously with some modifications^36^. Briefly, the bone marrow (BM) in the femur and tibia of the C57BL/6 mice were flushed with ice-cold 1X PBS. The BM cell suspension was collected by centrifugation at 500 × *g* for 10 min at room temperature. The red blood cells (RBCs) in the BM cell pellets were lysed by mixing the cell pellets with the RBC lysis buffer (0.14 M NH_4_Cl in 0.017 M Tris-HCl buffer, pH 7.6) for 10 min on ice^37^. The reaction was stopped by adding macrophage growth medium, and the residual BM cells were cultured in a 10-cm bacteriological dish (4 × 10^6^ cells/dish) in the presence of 10 ml macrophage growth medium supplemented with macrophage-colony stimulating factor (M-CSF, 20 ng/ml). An additional 5 ml of M-CSF-containing macrophage growth medium was added to the culture at day 3. At day 7 after culture, the non-adherent cells were removed by washing the cells with pre-warmed PBS, and the adherent BMMs were dissociated from the dish by incubating the cells with 5 mM EDTA. The suspended BMMs were washed once with 1X PBS and were resuspended in macrophage growth medium for further analysis. The animal experimental protocols were approved by the Chang Gung University Animal Care and Use Committee (approval ID: CGU15-034) and conducted in accordance with the policies and procedures described in the Guide for the Care and Use of Laboratory Animals National Research Council, National Academies Press, 1996.

### Lentivirus production

Lentiviruses were produced by the co-transfection of HEK-293T cells with 4 μg of short hairpin RNA (shRNA) lentiviral expression plasmid (mouse shDab2 clone B: 5′-CTCCAGTTACTTCAACAATAA-3′; and mouse shDab2 clone C: 5′-CCAGCAGTACAAGTCTGGAAT-3′), 4 μg of HIV-1 lentiviral packaging construct pCMVδ8.91 and 0.4 μg of VSV-G expression plasmid pMD.G using the Lipofectamine 2000 transfection method in 6-cm tissue culture plates. The lentiviral supernatant (3 ml) was harvested at 24 and 48 h post-transfection, respectively. After passing through a 0.22-μm filter, the supernatants were stored at −80 °C until use.

### Establishment of stable cell lines with a knockdown of Dab2 expression

RAW264.7 cells were plated at a density of 2 × 10^6^ cells/6-cm culture dish. At 24 h after cell plating, the cells were infected with lentiviral supernatant in the presence of 8 μg/ml of protamine sulfate. The culture medium was replaced with fresh DMEM medium 5 h post-infection. At 48 h post-infection, the cells were passaged into two 10-cm culture dishes containing 5 μg/ml of puromycin. Stable cell lines were used after at least 2 passages of puromycin selection.

### Microarray analysis

RAW264.7 cells (shLuc or shDab2-B) were treated with PBS or LPS (100 ng/ml) for 6 h. Total RNA was prepared by the TRI Reagent method^38^, and the RNA concentration was measured by spectrophotometer using a NanoDrop (Thermo Scientific, Wilmington, DE). The RNA integrity was verified using electrophoresis in a 1% agarose gel. RNA samples with an RNA integrity number greater than 8.0 underwent microarray analysis for profiling gene expression. Briefly, mouse whole genome OneArray microarray v2 (MOA-002) chips with 26,423 mouse genome probes and 872 experimental control probes were used (Phalanx Biotech Group). Two replicates were performed. All hybridized chips that met standard quality control criteria were calculated as the fold-changes. The data for microarray analysis were submitted to the GEO with the accession number GSE81088.

### Extraction of RNA and quantitative real-time RT-PCR

Two micrograms of total RNA was reverse transcribed using Superscript II (Invitrogen) and oligo dT primers. Quantitative real-time PCR was then performed using the LightCycler^®^ 480 instrument with the reaction conditions of pre-incubation at 95 °C for 10 min to activate HotStart Taq DNA polymerase, followed by 40 cycles of 15 s at 95 °C and 60 s at 65 °C. Water was used to replace cDNA in each run as a negative control in PCR amplification. The relative gene expression was determined by the ΔΔCt method^39^. The primer sets for PCR amplification are listed in Supplementary Table S1.

### Immunofluorescence staining

RAW264.7 cells were plated on the poly-L-lysine (Sigma) coverslips in 6-well plates at 5 × 10^5^/well. For immunofluorescence staining of Dab2, the cells were fixed with 4% paraformaldehyde for 30 min at room temperature, permeabilized with 0.1% Triton X-100 for 10 min and blocked with 3% bovine serum albumin/phosphate buffered saline (BSA/PBS) for 1 h. Cells were then incubated with the anti-Dab2 (p96) antibody (1:50) for 1 h at room temperature. After washing, the samples were incubated with the Alexa Fluor 488-conjugated anti-mouse IgG (1:200) and 4′,6-diamidino-2-phenylindole (DAPI) for 40 min. For the immunofluorescence staining of TLR4 and CHC, the cells were fixed in CellCover (Anacyte Laboratories, Hamburg, Germany) for 30 min at room temperature followed by blocking in 4% BSA/PBS for 30 min at room temperature. The biotinylated Sa15-21 (TLR4) and rabbit anti-CHC (1:250) antibodies were then added to the cells and incubated for 1 h at room temperature, followed by incubation with the Alexa Fluor 488-conjugated streptavidin (1:1000), Alexa Fluor 555-conjugated rabbit IgG (1:500) and DAPI for 40 min. The slides were mounted using fluorescent mounting medium (Dako, Hamburg, Germany) and were examined using a Zeiss LSM 510 confocal microscope (Carl Zeiss, Heidelberg, Germany). Images were acquired using the 63X oil objective. The co-localization coefficient between the indicated proteins was determined using the Zeiss LSM Image Browser Version (Carl Zeiss). Co-localization is defined as those areas where the fluorescent pixels of the two fluorescent channels overlay images that overlap. The co-localization coefficient is defined as the relative number of co-localizing pixels compared to the total number of pixels in the TLR4 above threshold.

### Enzyme-linked immunosorbent assay

The levels of cytokines for mouse TNF-α, IL-6 and RANTES were determined using the commercially available Ready-Set-Go (TNF-α and IL-6; eBioscience, San Diego, CA) and DuoSet (RANTES; R&D, Minneapolis, MN) ELISA kits. Briefly, cells were plated at a density of 2 × 10^5^ cells per well and starved for 2 h before stimulation with LPS. For the chlorpromazine (CPZ)-treated cells, cells were starved for 2 h with pre-treatment of 7.5 μM CPZ. After 2 h, cells were replaced with fresh serum-free DMEM followed by LPS stimulation. Supernatants were collected and assayed by ELISA according to the manufacturer’s instructions.

### Flow cytometry

For the immunodetection of cell surface expression of F4/80, a single-cell suspension (4 × 10^5^ cells/100 μl) in FACS buffer (0.1% BSA, 0.1% sodium azide in PBS) was incubated on ice for 10 min with the anti-CD16/CD32 antibody to block the Fc receptor. The cells were then incubated with the PE-Texas red-conjugated rat anti-mouse F4/80 antibody or the PE-Texas red-conjugated rat IgG2a κ isotype for 50 min. For analysis of TLR4/MD2 internalization, the single-cell suspension was incubated with the anti-CD16/CD32 antibody for 10 min on ice followed by incubation with the biotinylated rat anti-mouse TLR4/MD-2 monoclonal antibody MTS510 in FACS buffer. After several washes with the FACS buffer, the cells were incubated with the PE-conjugated streptavidin for 20 min and stained with 7-aminoactinomycin D to exclude the dead cells. The immunofluorescence signals were analyzed using a FACSCalibur system or C6 Accuri (BD Immunocytometry Systems).

### Cell surface biotinylation assay

The culture cells were washed twice with cold PBS before incubation with 0.2 mg/ml sulfo-NHS-SS-biotin (Thermo Fisher Scientific) in PBS/CM buffer (10 mM Na2HPO_4_, 2 mM KH_2_PO_4_, 137 mM NaCl, 2.7 mM KCl, 0.9 mM CaCl_2_ and 0.33 mM MgCl_2_) on ice for 30 min with shaking. The unbound biotin was quenched by adding 50 mM NH_4_Cl in PBS/CM buffer on ice for 10 min with gentle shaking. After cell surface labeling, the cells were transferred to the pre-warmed serum-free DMEM for 0, 15, 30 or 60 min at 37 °C to allow internalization of TLR4. At the indicated time, cells were washed twice with PBS/CM and then incubated three times with 100 mM 2-mercaptoethane sulfonic acid (MesNa) (Sigma) in a solution of 50 mM Tris-HCl, pH 8.6, 100 mM NaCl, and 2.5 mM CaCl_2_ for 10 min on ice to remove biotin. The free SH groups of MesNa were quenched by incubating the cells with 5 mg/ml iodoacetamide (Sigma) in PBS/CM on ice for 5 min. Cells were lysed in lysis buffer (50 mM HEPES, pH 7.5, 150 mM NaCl, 10% glycerol, 1.5% Triton X-100, 1.5 mM MgCl_2_, 10 μg/ml aprotinin, 10 μg/ml leupeptin, 1 mM PMSF, 200 μM sodium orthovanadate, and 100 μM EGTA). The lysates were clarified by centrifugation at 13,000 rpm for 10 min. The supernatants were incubated with 30 μl of NeutrAvidin-agarose (Pierce, IL, USA) at 4 °C overnight, and the immunoprecipitates were analyzed by Western blotting using the anti-TLR4 antibody (Cell Signaling, Beberly, MA). Image J analysis software was used for quantification of the changes in TLR4 internalization.

### Preparation of cell lysates, Western blot analysis and immunoprecipitation

For the preparation of cell lysates, the culture cells were washed twice and lysed in the lysis buffer for 30 min on ice. After centrifugation at 13,000 rpm for 10 min, the supernatants were collected as the total cell lysates. Western blot analysis was performed as described previously^40^. Briefly, cell lysates were fractionated on SDS-PAGE, and the proteins were transferred onto a polyvinylidene difluoride (PVDF) membrane. After blocking the membrane with 5% non-fat dry milk in 1X PBS containing 0.1% Tween 20 (PBST) at room temperature or in blocking buffer (T-PRO, Taiwan) overnight at 4 °C (for p-IRF3 detection), the membrane was incubated with the indicated primary antibodies and subsequently the horseradish peroxidase (HRP)-conjugated anti-mouse or anti-rabbit secondary antibodies (1:10000). Specific protein bands were detected using the chemiluminescent HRP substrate as described by the manufacturer (Millipore). The concentrations of the primary antibodies used in the Western blotting were 1:3000 for anti-Dab2 (p96), 1:10000 for anti-β-actin, 1:1000 for anti-phopho-IRF3 (Ser396), 1:3000 for anti-IRF3, 1:1000 for anti-IRAK1, 1:3000 for anti-IκB-α, 1:3000 for anti-phospho-p65 (Ser536), 1:1000 for anti-phospho-p38, 1:1000 for anti-phospho-ERK1/2, 1:1000 for anti-phospho-JNK, 1:3000 for anti-CHC, and 1:1000 for anti-TLR4 (D8L5W).

For immunoprecipitation analysis, the proteins were pre-cleared by incubation of the cell lysates with 15 μl of protein G agarose (Millipore) beads for 1 h at 4 °C. The cell lysates were then incubated with 2 μg of anti-Dab2 (H-110) (Santa Cruz) antibody in the presence of 30 μl of protein G agarose at 4 °C for 2.5 h. The immuno-precipitated lysates were electrophoresed on 10% SDS-PAGE gels, and the fractionated proteins were subsequently transferred to the PVDF membrane. The membrane was blocked with 5% nonfat milk and probed with the appropriate antibodies.

### Statistical analysis

Statistical analysis was performed using Prism software, version 5 (GraphPad Software). A 2-tailed, unpaired Student’s *t* test was used. A *p* value of less than 0.05 was considered statistically significant.

## Results

### Dab2 expression in murine macrophages

**Figure 1:**
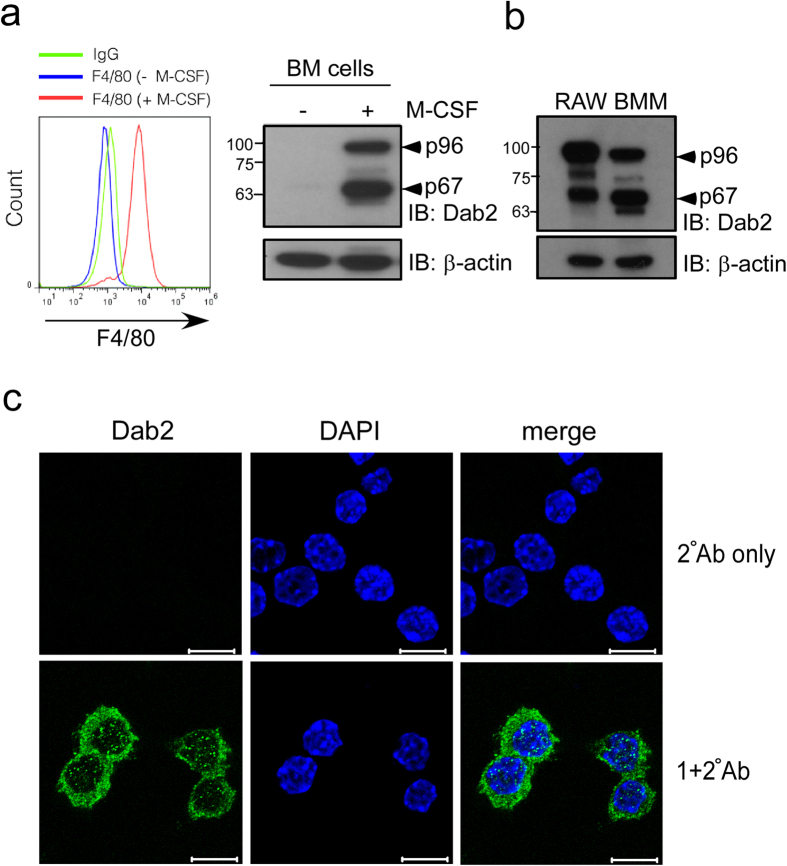
Dab2 expression in murine macrophages. **(a,b)** Murine BM cells were obtained from mouse femur and tibia and were subject to *ex vivo* differentiation in the presence or absence of M-CSF. BMM were collected and analyzed by flow cytometry using the anti-F4/80 antibody (panel a, left). The lysates of BMM (panel a, right) and RAW264.7 (panel b) cells were analyzed by Western blotting using the anti-Dab2 (p96) antibody. The expression of β-actin was used for the control of equal protein loading. The full Western blot and the corresponding positions of the molecular weight protein markers are presented in Supplementary Fig. S2. **(c)** Dab2 localization in the RAW264.7 cells was analyzed by immunofluorescent staining followed by confocal microscopy analysis. Immunofluorescent staining was performed using the mouse anti-Dab2 (p96) primary antibody followed by incubation with the Alexa Fluor 488-conjugated anti-mouse IgG secondary antibody (green). Secondary antibody staining alone was represented as a negative control of fluorescent signal (upper panel). Nucleated cells were defined by positive fluorescent staining of DAPI (blue). Scale bar: 10 μm.

Dab2 is predominantly expressed in inflammatory macrophages recruited to the injured rat brain^41^. Dab2 was not detectable in murine BM cells. p96 (also known as p82) and p67 (also known as p59) isoforms of Dab2 were expressed in the *ex vivo* differentiated murine BMM that were positive for the surface marker F4/80 (Fig. 1a). Both isoforms of Dab2 were also expressed in the murine macrophage-like RAW264.7 cells with a cytoplasmic punctuate expression pattern that was consistent with the endocytic function of Dab2 (Fig. 1b,c). These data indicate that BMM and RAW264.7 cells expressed both isoforms of Dab2.

### Dab2 regulates the TLR4/TRIF-dependent inflammatory response

**Figure 2:**
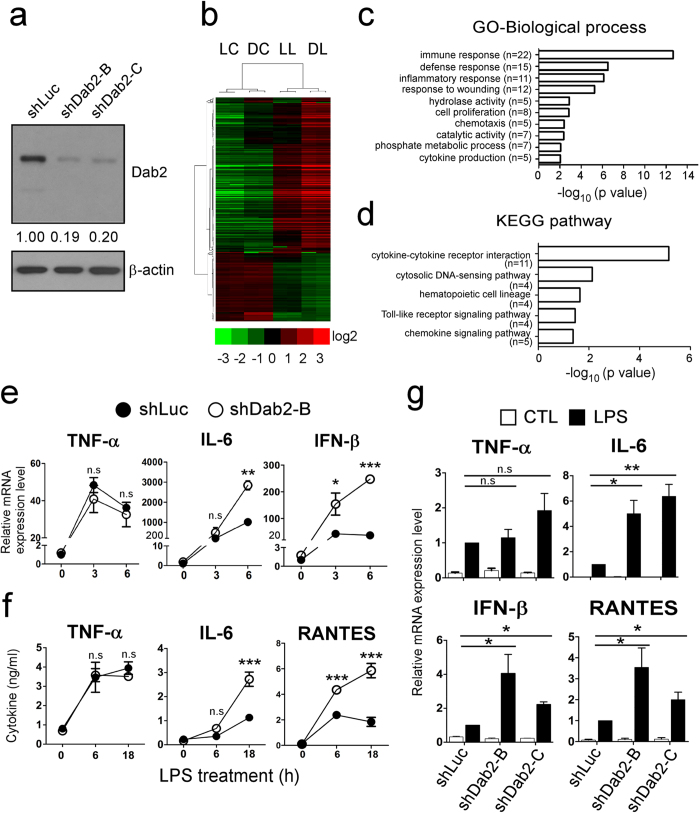
Dab2 regulates LPS-induced TLR4/TRIF-dependent inflammatory response. (**a**) Dab2 expression in the shLuc, shDab2-B and shDab2-C cells was determined by Western blotting using the anti-Dab2 (p96) antibody. The expression of β-actin was used as a control of equal protein. The relative expression level of Dab2 after normalization by the expression of β-actin is shown. The full Western blot and the corresponding positions of the molecular weight protein markers are presented in Supplementary Fig. S2. (**b**) The total RNAs obtained from the shLuc and shDab2-B cells with or without LPS treatment for 6 h were subject to microarray analysis. The 926 normalized genes that were differentially expressed by treatment with LPS (LL *vs.* LC, and DL *vs.* DC) or by knockdown of Dab2 (DC *vs.* LC, or DL *vs.* LL) with the Log_2_ ratio >1.5 or <−1.5 are shown in the heat map. LC, shLuc cells without LPS treatment; LL, shLuc cells with LPS treatment; DC, shDab2-B cells without LPS treatment; and DL, shDab2-B cells with LPS treatment. **(c,d)** Significant gene ontology enrichment analysis related to biological process **(c)** and KEGG pathway **(d)** ordered according to –log (p-value) **(e)** The shLuc and shDab2-B cells were treated with LPS (100 ng/ml) for 3 and 6 h. The mRNA expression of pro-inflammatory mediators was determined by real-time RT-PCR and was normalized by the expression of *gapdh*. The levels of each cytokine mRNA expression for the shLuc cells without LPS treatment were arbitrarily set as 1. Data represent the mean ± SEM of three independent experiments. **(f)** The shLuc and shDab2-B cells were treated with LPS (100 ng/ml) for the indicated time. The culture medium was collected for ELISA assays to measure the secreted levels of the indicated pro-inflammatory mediators. Data represent the mean ± SEM of three independent experiments. **(g)** The shLuc, shDab2-B and shDab2-C cells were treated with LPS (100 ng/ml) for 6 h. Total RNAs were isolated from the indicated cell lines for real-time RT-PCR analyses of the indicated pro-inflammatory mediators. Data represent the mean ± SEM for the fold increase of the pro-inflammatory mediators mRNA in shDab2-B or shDab2-C cells when compared with the LPS-treated shLuc cells in a representative experiment. At least four independent experiments were performed. n.s., no significance. *P < 0.05; **P < 0.01; ***P < 0.001; and ****P < 0.0001.

RAW264.7 cells with a stable knockdown of Dab2 expression were established by infecting the cells with lentiviruses encoding the shRNA of Dab2 (B and C clones) to investigate whether Dab2 plays a role in the inflammatory response of macrophages. The stable cell line expressing shRNA of luciferase (shLuc) was used as a control. The expression of the Dab2 protein in the shDab2-B and shDab2-C cells was decreased to approximately 20% of the control shLuc cells as revealed by Western blot analysis (Fig. 2a). The shLuc and shDab2-B cells stimulated with LPS (LL and DL group) or the vehicle control (LC and DC group) were subject to global profiling of gene expression using the Affymetrix mouse OneArray chip. The genes that were differentially expressed by treatment with LPS (LL *vs.* LC, and DL *vs.* DC) or by the knockdown of Dab2 (DC *vs.* LC, or DL *vs.* LL) with the Log_2_ ratio >1.5 or <−1.5 are shown in the clustering analysis (Fig. 2b). A vast number of genes in shLuc and shDab2-B cells, including the innate immune response genes encoding cytokines, chemokines, signaling molecules, and transcriptional factors, were up-regulated or down-regulated by treatment with LPS (Supplementary Tables S2 and S3; LL *vs.* LC, and DL *vs.* DC). When compared to shLuc control cells, knockdown of Dab2 caused an up-regulation of 34 genes and a down-regulation of 18 genes in the absence of LPS (Supplementary Table S4, DC *vs.* LC), while it resulted in an up-regulation of 104 genes and a down-regulation of 32 genes in the presence of LPS (Supplementary Table S5, DL *vs.* LL).

The GO enrichment and KEGG pathway analyses were performed to organize the biological processes (Fig. 2c) and pathways (Fig. 2d) that were differentially activated between shLuc and shDab2 cells upon LPS treatment. In the biological process, the most significant GO term was ‘immune response’ with 22 transcripts enriched, following by GO term related to defense response, inflammatory response, response to wounding, hydrolase activity, cell proliferation, chemotaxis, catalytic activity, phosphate metabolic process and cytokine production (Fig. 2c). Most of them were likely responsible for the hyperactive cytokine and chemokine production of shDab2 cells. On the other hand, KEGG pathway analysis revealed the five most significant canonical pathways were cytokine-cytokine receptor interaction, cytosolic DNA-sensing pathway, hematopoietic cell lineage, Toll-like receptor signaling pathway and chemokine signaling pathway (Fig. 2d). These data imply that knockdown of Dab2 affects mainly the inflammatory responses induced by LPS stimulation.

LPS-induced TLR4 signaling is classified into MyD88-dependent and TRIF-dependent pathways^42^. The differentially expressed genes between LPS-treated shDab2-B and shLuc cells (DL *vs*. LL) were compared with the LPS-inducible genes to clarify the effects of Dab2 knockdown on MyD88- and TRIF-dependent gene expression. Attenuated Dab2 expression resulted in the up-regulation and the down-regulation of the MyD88-dependent genes *Pilra* and *Adssl1*, respectively (Table 1). Other genes related to the MyD88-dependent pathway, such as the pro-inflammatory cytokines *Tnf* and *Il1b*, the chemokines *Cxcl2/Mip-2a* and *Ccl3/Mip-1a*, the intracellular signaling molecule *Socs3*, and the enzymes *Ptgs2/Cox-2* and *Pde4b*, were not affected by the knockdown of Dab2 (data not shown). The expression of MyD88/TRIF-dependent pro-inflammatory cytokines *Il6* and *Il1a*, the TRIF-dependent type I IFN *Ifnb*, and the IFN-inducible genes *Cxcl10*, *Ifit1*, *Ccl5* and *Ifit2* were up-regulated by the knockdown of Dab2 (Table 1). These data indicate that Dab2 is mainly involved in the regulation of TRIF-dependent gene expression with a slight to moderate effect on MyD88-dependent gene expression in response to LPS stimulation.

The shLuc and shDab2-B cells were stimulated by LPS, and the expression of representative cytokines/chemokines downstream of the MyD88- and TRIF-dependent pathway were analyzed by RT-qPCR to confirm the data obtained by microarray analysis. The MyD88-dependent expression of *Tnf* mRNA peaked at 3 h after LPS stimulation with no significant difference between shLuc and shDab2-B cells (Fig. 2e, p = 0.11). The shDab2-B cells showed a 2.7-fold (p < 0.01) and 6.8-fold (p < 0.001) increase in the LPS-stimulated expression of *Il6* and *Ifnb* mRNA, respectively, when compared to shLuc cells. Consistent with the mRNA analysis, the secreted TNF-α protein peaked at 6–18 h post-LPS treatment with no significant difference between shLuc and shDab2-B cells (Fig. 2f, p = 0.83 at 6 h, p = 0.12 at 18 h). When compared to shLuc cells, shDab2-B cells had a 2.3-fold (p < 0.001) and a 5.8-fold (p < 0.001) increase in the levels of secreted IL-6 and RANTES proteins, respectively, at 18 h after LPS treatment (Fig. 2f). These data confirmed that the knockdown of Dab2 augments the TRIF-dependent inflammatory response.

To exclude possible off-target effects of shRNA, another shRNA targeting of a different sequence of Dab2 was used to establish an additional stable subline of shDab2 (shDab2-C). The shLuc control cells and the Dab2 knockdown cells shDab2-B and shDab2-C showed no difference (p = 0.42 for shDab2-B; p = 0.16 for shDab2-C) in the expression of *Tnf* mRNA 6 h after LPS treatment. shDab2-B and shDab2-C cells had a higher expression of *Il6* (p < 0.01 for shDab2-B and p < 0.001 for shDab2-C)*, Ifnb* (p < 0.05 for shDab2-B and p < 0.01 for shDab2-C) and *ccl5 (Rantes*, p < 0.05 for shDab2-B and shDab2-C) mRNA when compared with the shLuc cells (Fig. 2g). These data rule out the potential off-target effect of shDab2 on cytokine/chemokine gene expression.

### Dab2 functions as a negative regulator of LPS-stimulated TLR4/MD2 internalization and IRF3 activation

**Figure 3:**
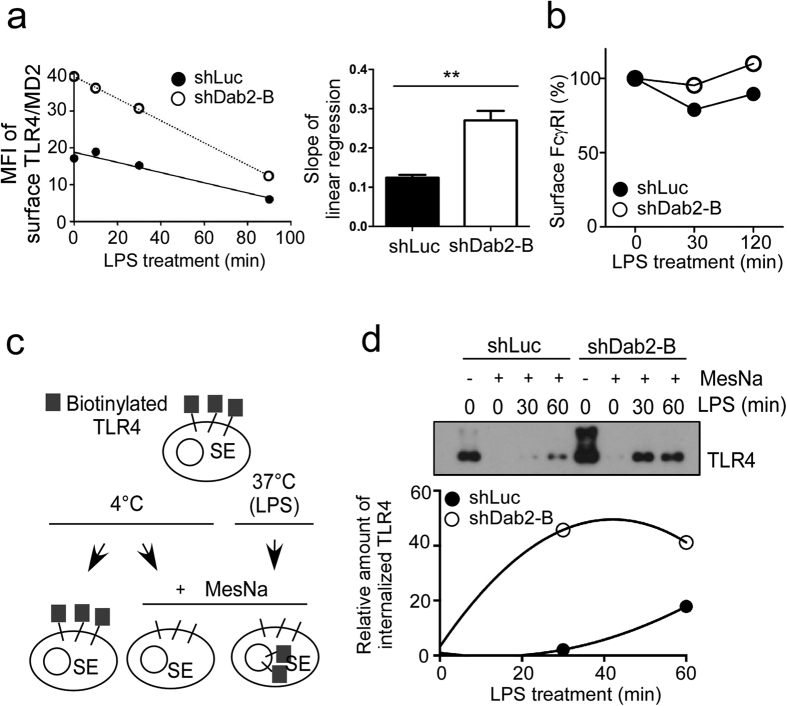
Loss of Dab2 accelerates LPS-stimulated TLR4/MD2 internalization. **(a,b)** shLuc and shDab2-B cells were treated with LPS (100 ng/ml) for the indicated time. Flow cytometry was then performed to examine the surface levels of TLR4/MD2 complex and, as a negative control, FcγRI. The expression levels of the surface TLR4/MD2 complex (panel A, left) and FcγRI (panel B) were plotted. The slope for linear regression of the data representing the rate for TLR4/MD2 internalization was determined (panel A, right). Data represent the mean ± SEM of three independent experiments. **P < 0.01. **(c)** Schematic representation of the surface protein biotinylation analysis for quantitative assessment of TLR4 internalization. (**d)** The shLuc and shDab2-B cells treated with LPS (100 ng/ml) for the indicated time were subject to the surface protein biotinylation assay. The internalized proteins in the lysates were pulled down and analyzed by Western blotting using the anti-TLR4 antibody. Quantification for the changes of internalized TLR4 was performed by using the Image J analysis software. The relative levels of internalized TLR4 in shLuc or shDab2-B cells when compared with the MesNa-treated vehicle shLuc cells in a representative experiment are shown. The full Western blot and the corresponding positions of the molecular weight protein markers are presented in Supplementary Fig. S2.

Dab2 is an adaptor protein involved in receptor-mediated endocytosis^32^. To determine whether Dab2 regulated LPS-stimulated TLR4/MD2 internalization, the levels of TLR4/MD-2 complex remaining on the cytoplasmic membrane of the LPS-treated shLuc and shDab2-B cells was examined by flow cytometry using an antibody that recognizes the TLR4/MD2 complex (MTS510 antibody). A 2.18-fold increase in the cell surface TLR4/MD2 expression was noticed for shDab2-B cells when compared to shLuc cells in the resting stage. The TLR4/MD2 complexes for both cell lines steadily decreased in a time-dependent manner and were almost undetectable at 90 min after LPS stimulation (Fig. 3a, left panel). The slope of the linear regression line, which represents the internalization rate of TLR4/MD2 during LPS treatment, was −0.12 ± 0.01 (n = 3) and −0.27 ± 0.02 (n = 3) for shLuc and shDab2-B cells, respectively (Fig. 3a, right panel, p < 0.01). Knockdown of Dab2 expression did not affect the internalization of the receptor FcγRI, which is not associated with LPS signaling (Fig. 3b). These data indicate that Dab2 regulates LPS-induced internalization of the TLR4/MD2 complex.

The cell surface biotinylation assay was performed to confirm the effects of Dab2 knockdown on LPS-induced TLR4/MD2 internalization. The surface proteins of shLuc and shDab2-B cells were labeled with the non-permeable but reversible biotinylation reagent sulfo-NHS-SS-biotin at 4 °C followed by warming to 37 °C in the presence of LPS for the indicated time (Fig. 3c). Cells were then returned to 4 °C and treated with the reducing agent MesNa to remove biotin from recycling protein or non-internalized receptors. Biotinylated TLR4 was immunoprecipitated by neutravidin agarose beads and was analyzed using Western blotting. This method allowed quantification of internalized TLR4 without interference of the TLR4 on the cell surface. Our data revealed that LPS-induced TLR4 internalization in a time-dependent manner. The amount of internalized TLR4 at 30 and 60 min after LPS treatment was approximately 21.6-fold and 2.3-fold greater in the shDab2-B cells when compared to shLuc cells, respectively (Fig. 3d).

**Figure 4:**
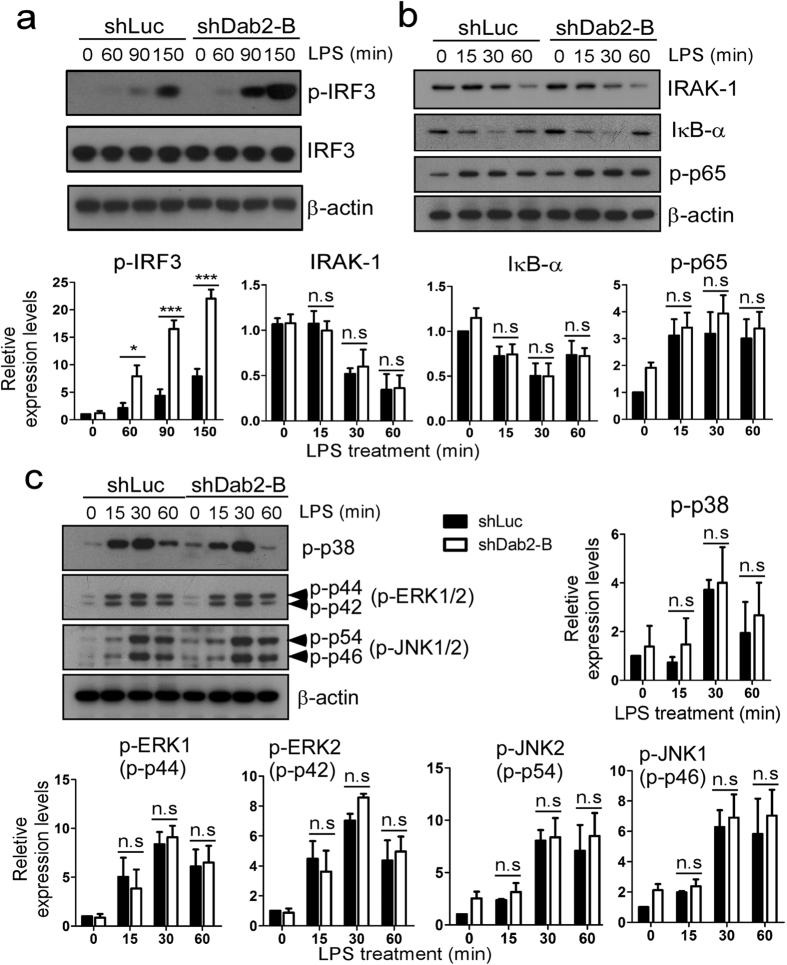
The TRIF-dependent phosphorylation of IRF-3 is augmented in Dab2-knockdown cells. **(a–c)** The shLuc and shDab2-B cells were treated with LPS (100 ng/ml) for the indicated time. The cell lysates were collected for Western blotting using the antibodies against the indicated proteins to determine the effect of Dab2 knockdown on LPS-stimulated phosphorylation of IRF-3 (panel A), p65-NF-κB (panel B), and MAPKs (p38, ERK1/2, and JNK1/2; panel C), and degradation of IRAK1 and IκBα (panel B). The expression of β-actin was used as a control for equal protein loading. Quantification for the changes of protein phosphorylation and degradation was performed by using the Image J analysis software. The full Western blot and the corresponding positions of the molecular weight protein markers are presented in Supplementary Fig. S3. Data represent the mean ± SEM of three independent experiments. ns, no significance. *P < 0.05; **P < 0.01; and ***P < 0.001.

LPS induces TLR4 internalization to form endosomes, playing an essential role in the TRIF-dependent phosphorylation/activation of IRF3^12^,^43^. The phosphorylation status of IRF3 was increased in the shDab2-B cells by 2.8-fold at 150 min post-LPS treatment when compared to shLuc cells (Fig. 4a, p < 0.001). LPS induced MyD88-dependent degradation of IRAK1 and IκBα and the phosphorylation of p65-NFκB and MAPKs (p38, ERK1/2 and JNK1/2) in a time-dependent manner with no difference between shLuc and shDab2-B cells (Fig. 4b,c). This indicates that Dab2 underlies LPS-stimulated TRIF-dependent signaling and selectively regulates TLR4/MD2 internalization and IRF3 activation in murine macrophages.

### Dab2 sequesters clathrin to prevent excessive TLR4 internalization and TRIF-dependent signaling

**Figure 5:**
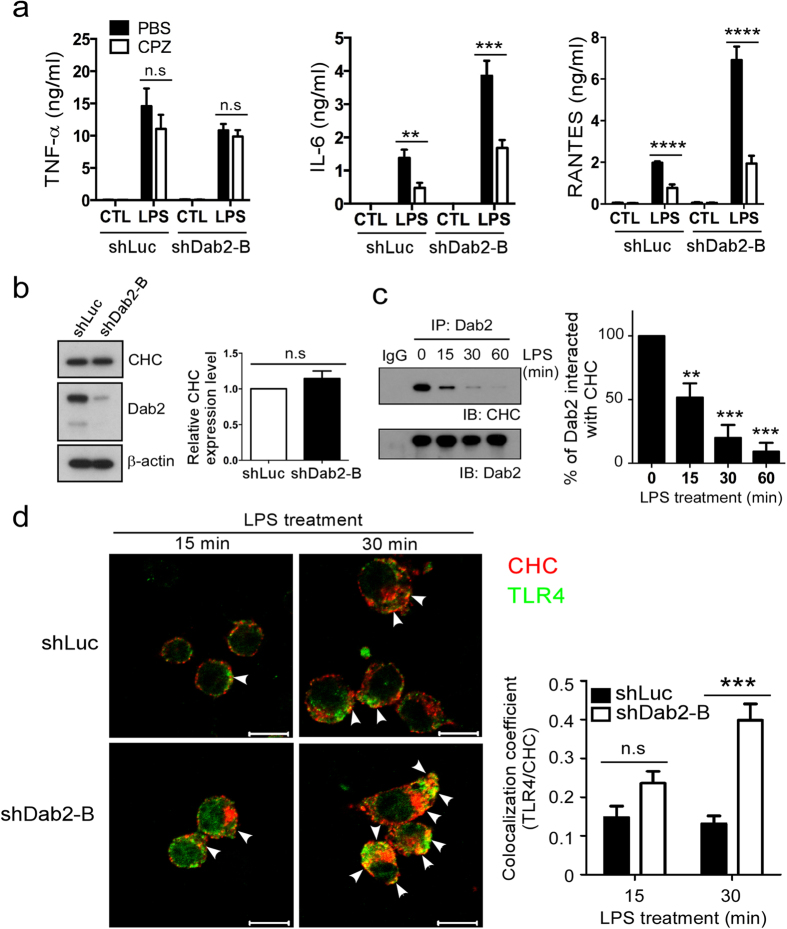
Dab2 interacts with clathrin and regulates the association of TLR4 with clathrin. **(a)** The shLuc and shDab2-B cells were pre-treated with 7.5 μM CPZ for 30 min followed by LPS stimulation for 18 h. The levels of the indicated cytokines/chemokines present in the culture medium were measured by ELISA. Data represent the mean ± SEM of three independent experiments. **(b)** The lysates of shLuc and shDab2-B cells were subject to Western blotting using the anti-CHC antibody. The expression of β-actin was used for the control of equal protein loading. The relative expression levels of CHC after normalization by the expression of β-actin are shown. The full Western blot and the corresponding positions of the molecular weight protein markers are presented in Supplementary Fig. S4. **(c)** The shLuc cells were treated with LPS (100 ng/ml) for the indicated time. The cell lysates were collected for immunoprecipitation of Dab2 using the anti-Dab2 (H-110) antibody. The immuno-precipitated lysates were subject to Western blotting using the anti-CHC antibody. Data represent the mean ± SEM of three independent experiments. The full Western blot and the corresponding positions of the molecular weight protein markers are presented in Supplementary Fig. S4. **(d)** The shLuc and shDab2-B cells were treated with LPS for 15 and 30 min. Immunofluorescence staining was then performed using the rabbit anti-mouse CHC (red) and biotinylated anti-TLR4 (green) primary antibodies followed by the alexa fluor 555-conjugated donkey anti-rabbit IgG antibody and alexa fluor 488-conjugated streptavidin. The co-localization of TLR4 and CHC was determined by using Zeiss LSM Image Browser Version. Co-localization was determined by use of the co-localization coefficient between 15–30 cells in each group. ns, no significance. *P < 0.05; **P < 0.01; ***P < 0.001, and ****P < 0.0001. Scale bar: 10 μm.

LPS-induced TLR4 internalization and transport to the endosome is a clathrin-dependent process^14^. The clathrin assembly inhibitor CPZ had no effect on LPS-stimulated TNF-α expression in shLuc (p = 0.76) and shDab2-B cells (p = 0.52) (Fig. 5a). In contrast, CPZ inhibited not only LPS-stimulated IL-6 (p < 0.01) and RANTES (p < 0.0001) in the shLuc cells but also the excessive production of IL-6 (p < 0.001) and RANTES (p < 0.0001) in the LPS-treated shDab2-B cells. These data indicate that clathrin-mediated endocytosis contributes to the increased release of IL-6 and RANTES caused by Dab2 knockdown.

The following studies were performed to elucidate how Dab2 controls the LPS-stimulated internalization of TLR4. Clathrin heavy chain (CHC) protein is the key subunit of clathrin and plays a role in clathrin-mediated endocytosis by mediating the interactions of clathrin-coated vehicles with various downstream endocytic adaptors^44^,^45^. The shDab2-B cells had similar levels of CHC expression when compared to the shLuc cells, implying that aberrant CHC expression did not account for the effects of Dab2 on LPS-induced TLR4 internalization (Fig. 5b). Co-immunoprecipitation analysis of shLuc cell lysates revealed that Dab2 interacted with clathrin in the resting stage of macrophages. The level of interaction was reduced to 51 ± 9% and 9 ± 6% of the resting control cells, respectively, at 15 and 60 min after LPS treatment (Fig. 5c). Confocal microscopy was performed to define whether Dab2 regulates the co-localization of TLR4 and CHC in the LPS-treated shLuc and shDab2-B cells. TLR4/CHC co-localization was observed at 15 min (coefficient: 0.14 ± 0.02) and 30 min (coefficient: 0.13 ± 0.01) after stimulation of the shLuc cells with LPS (Fig. 5d, upper panel). The co-localization of TLR4 and CHC in the shDab2-B cells was increased 2-fold (coefficient: 0.30 ± 0.01, p = 0.06) and 3-fold (coefficient: 0.40 ± 0.02, p < 0.001) at 15 and 30 min post-LPS treatment when compared to shLuc cells, respectively (Fig. 5d, lower panel). These data indicate that Dab2 regulates the association of clathrin with TLR4 in LPS-stimulated macrophages.

## Discussion

**Figure 6:**
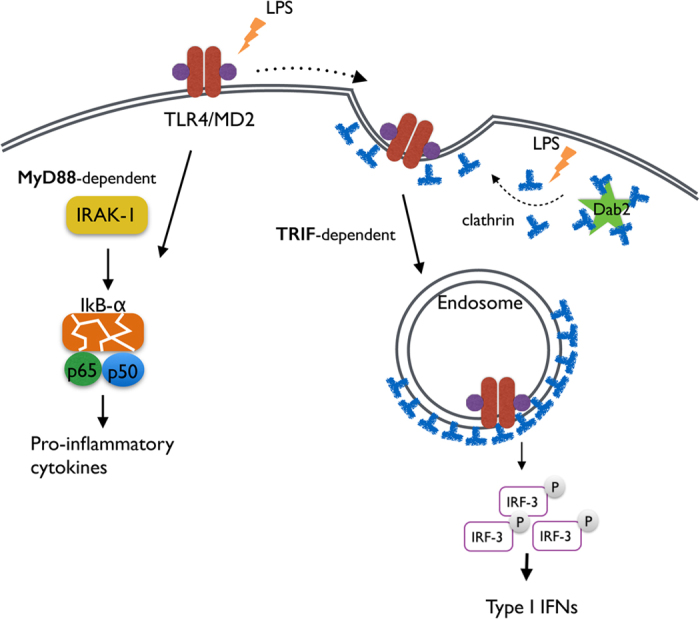
A Schematic diagram of TLR4 endocytic regulation by Dab2. In the steady state, Dab2 sequesters clathrin from the TLR4/MD2 complex. TLR4 signals via two pathways in response to LPS. The surface TLR4 signal via MyD88 activates NF-κB and pro-inflammatory cytokine production. Clathrin is released from Dab2 and is associated with TLR4 upon LPS stimulation. TLR4/MD2 then undergoes endocytosis in a clathrin-dependent manner resulting in IRF3 phosphorylation and type I interferon production.

The internalization of TLR4/MD2 upon LPS stimulation is important for macrophage function. Dab2 is a negative regulator of the internalization of TLR4/MD2 by sequestering clathrin and restricting the interaction of TLR4 with clathrin. As a result, Dab2 controls the extent of TRIF-dependent IRF-3 activation and the expression of type I interferon production (Fig. 6). This study highlights the novel functions of Dab2 in the inflammatory response of macrophages to LPS.

TLR4 signaling and its internalization in macrophages is one of the central themes in innate immunity. The signaling pathways following TLR4 activation have been extensively studied^11^,^46^,^47^. In addition to the core signal proteins underlying the TRIF- and MyD88-pathways, IRAK-M, PDCD4, CD11b and SHP act as negative regulators^48^,^49^,^50^,^51^, whereas Pin1, p110δ and CD14 act as positive regulators^15^,^47^,^52^ of TLR4 signaling. However, only a few accessory molecules have been reported to regulate TLR4 internalization and intracellular trafficking. TMED7 and Rab10 regulate TLR4 trafficking to the cell surface and/or to the late endosome^18^,^53^. Rab7b directs the degradation of TLR4 in lysosomes^54^. Phospholipase Cγ-2 and CD14 promote LPS-induced endocytosis of activated TLR4^15^. Dab2 regulates receptor trafficking and cell signaling in various tissues and cell types^25^,^27^,^38^,^55^. In this study, Dab2 was defined as a negative regulator of LPS-induced TLR4 signaling and trafficking in macrophages in addition to playing a role in the control of inflammatory signaling during phenotypic polarization, spreading and adhesion of macrophages^34^,^56^.

Dab2 negatively regulates MyD88-dependent inflammatory cytokine mRNA expression, such as *Il1b*, *Ptgs2* (COX2), *Il6* and *Tnfa*, in response to TLR2 and TLR4 agonists in macrophages^56^. No mention is made in that study regarding whether or not Dab2 has any effect on LPS-stimulated activation of the TRIF signaling pathway. In the current study, Dab2 was shown to regulate IRF3 activation and gene expression of type I interferon. Although LPS-induced MyD88-dependent expression of *Il1b* and *Ptgs2* was up-regulated in the Dab2-silenced cells, their levels of expression did not fulfill the criteria considered for up-regulation or down-regulation (Log_2_ >1.5 or <−1.5). These studies suggest that Dab2 plays dual roles in LPS signaling by contributing mainly to the regulation of the TRIF-dependent pathway with a moderate effect on the MyD88-dependent pathway.

Different internalization signals and cargo contents mediate endocytosis of the receptors and their bound ligands^57^. Dab2 is mainly known as an adaptor protein facilitating ligand-induced endocytosis of receptors with the NPXY motif but not receptors with YXXØ sequences^32^,^58^. However, Dab2 elicits cell type and cell culture condition-specific effects on the internalization of transferrin-bound transferrin receptor, which is a YXXØ type endocytic cargo^38^. Dab2 may be linked to the reduced internalization of NPXY type endocytic cargos concomitant with an increased internalization of the YXXØ type endocytic cargos in the 2-methoxyestradiol (2-ME2)-treated ES-2 ovary cancer cells arrested in the spindle assembly checkpoint of the G2/M phase^59^. The current study supports a new Dab2 function in LPS-induced internalization of TLR4 that belongs to the type of endocytic cargo with the YXXØ internalization signal. Because ligand-induced TLR4 internalization has a role in signal transmission and down-regulation of the cell surface levels of receptor^18^,^47^, it is implied from the results of the current study that Dab2 is functionally important in maintaining the homeostasis of TLR4 signaling.

According to our study, Dab2 forms a protein complex with clathrin in the resting stage of macrophages. The interaction between Dab2 and clathrin was diminished after LPS treatment, followed by an increase in the co-localization of clathrin and TLR4, ultimately facilitating LPS-induced TLR4/MD2 internalization. Dab2 is not likely to interact directly with TLR4 to mediate receptor internalization because TLR4 does not have a putative binding sequence for Dab2 (data not shown). Instead, Dab2 releases clathrin from the Dab2-clathrin complex to allow for the formation of clathrin-coated pits at the cytoplasmic tail of TLR4 upon LPS stimulation. This mode of action is supported by several findings, as shown in this and other studies. Knockdown of Dab2 accelerates the internalization rate of TLR4 and increases the amount of internalized TLR4 following stimulation of macrophages by LPS. The concept of a “clathrin sponge” has been proposed previously on the basis of the findings that huntingtin-interacting protein 1, which specifically binds clathrin, sequesters clathrin and acts as a clathrin sponge to prevent endocytosis of activated receptor tyrosine kinases to form endosomes in cancers^60^. Dab2 interacts with clathrin, and the interaction is diminished during mitosis, causing an increased internalization of the YXXØ type endocytic cargos^59^. Based on these findings, we proposed that Dab2 may act as a “clathrin sponge” in the resting stage of macrophages. Clathrin released from the Dab2-clathrin protein complexes mediates TLR4 internalization when the macrophages are stimulated by LPS. The detailed mechanism for the dissociation of Dab2 and clathrin is not yet clear. However, we have observed that LPS treatment of the RAW264.7 cells caused mobility retardation of Dab2 protein when the cell lysates were fractionated on SDS-PAGE. The mobility retardation can be reversed by treatment with calf intestine phosphatase, indicating Dab2 was phosphorylated upon LPS stimulation (Supplementary Fig. S1). It has been reported that treatment of ES-2 ovary cancer cells by 2-methoxyestradiol (2ME2) induces cell cycle arrest in G2/M phase and Dab2 phosphorylation accompanied with gradual dissociation of Dab2 from the cytoplasmic membrane and a loss of co-localization with clathrin. Mutation of the mitotic phosphorylation sites of Dab2 reduces its cytoplasmic membrane dissociation upon 2ME2 treatment, implying that Dab2 phosphorylation reduces its interaction with cytoplasmic membrane and clathrin^59^. Whether LPS-induced Dab2 phosphorylation relates to the dissociation between Dab2 and clathrin is worthy to investigate further.

Dab2 is a new regulator of innate immunity through the regulation of LPS-induced TLR4/TRIF endocytic signaling. The newly identified link between Dab2 and the TRIF-dependent inflammatory response opens additional avenues to explore the immunological function of Dab2 and the therapeutic targets of endotoxin-induced sepsis.

## Additional Information

**How to cite this article**: Hung, W.-S. *et al*. Disabled-2 is a negative immune regulator of lipopolysaccharide-stimulated Toll-like receptor 4 internalization and signaling. *Sci. Rep.* **6**, 35343; doi: 10.1038/srep35343 (2016).

## Supplementary Material

Supplementary Information

## Figures and Tables

**Table 1:** The differentially expressed MyD88- and TRIF-dependent genes between LPS-treated shLuc and shDab2-B cells.

GenBank Accession No.	Gene Symbol	Log_2_ Ratio	Fold Change
**MyD88-dependent up-regulated gene (DL/LL)**			
NM_153510.3	*Pilra*	2.1	4.4
**MyD88-dependent down-regulated gene (DL/LL)**			
NM_007421.2	*Adssl1*	−1.6	−3.1
**MyD88/TRIF-dependent up-regulated genes (DL/LL)**			
NM_031168.1	*Il6*	4.6	24.4
NM_010554.4	*Il1a*	2.4	5.1
**TRIF-dependent up-regulated genes (DL/LL)**			
NM_010510.1	*Ifnb*	2.3	5.1
NM_021274.2	*Cxcl10*	2.1	4.4
NM_008331.3	*Ifit1*	2.0	4.0
NM_013653.3	*Ccl5*	1.5	2.7
NM_008332.3	*Ifit2*	1.5	2.7
