# Supplementary material for: Disabled-2 is a negative immune regulator of lipopolysaccharide-stimulated Toll-like receptor 4 internalization and signaling
Source: Sci Rep. 2016 Oct 17;6:35343. doi: 10.1038/srep35343 (PMC5066213; doi:10.1038/srep35343)
Supplement: Supplementary Information [file srep35343-s1.pdf]

Disabled-2 is a negative immune regulator of lipopolysaccharide-stimulated Toll-like receptor 4 internalization and signaling

Wei-Shan Hung[1], Pin Ling[2,3], Ju-Chien Cheng[4], Shy-Shin Chang[5], Ching-Ping Tseng[1,6,7,8]

[1]Graduate Institute of Biomedical Sciences, College of Medicine, Chang Gung University, Kwei-Shan, Taoyuan 333, Taiwan, Republic of China

[2]Institute of Basic Medical Sciences, College of Medicine, National Cheng Kung University, Tainan 701, Taiwan, Republic of China

[3]Department of Microbiology and Immunology, College of Medicine, National Cheng Kung University, Tainan 701, Taiwan, Republic of China

[4]Department of Medical Laboratory Science and Biotechnology, China Medical University, Taichung 404, Taiwan, Republic of China

[5]Department of Family Medicine, Chang Gung Memorial Hospital, Kweishan, Taoyuan 333, Taiwan, Republic of China

[6]Department of Medical Biotechnology and Laboratory Science, College of Medicine, Chang Gung University, Kwei-Shan, Taoyuan 333, Taiwan, Republic of China

[7]Molecular Medicine Research Center, Chang Gung University, Kwei-Shan, Taoyuan 333, Taiwan, Republic of China

[8]Department of Laboratory Medicine, Chang Gung Memorial Hospital, Kwei-Shan, Taoyuan 333 , Taiwan, Republic of China

To whom all correspondence should be addressed:

Ching-Ping Tseng, Department of Medical Biotechnology and Laboratory Science, College of Medicine, Chang Gung University, Kwei-Shan, Taoyuan 333, Taiwan, Republic of China. Tel.: +886-3-2118800 ext. 5202; Fax: +886-3-2118355; e-mail: ctseng@mail.cgu.edu.tw.

Supplementary Figure and Tables

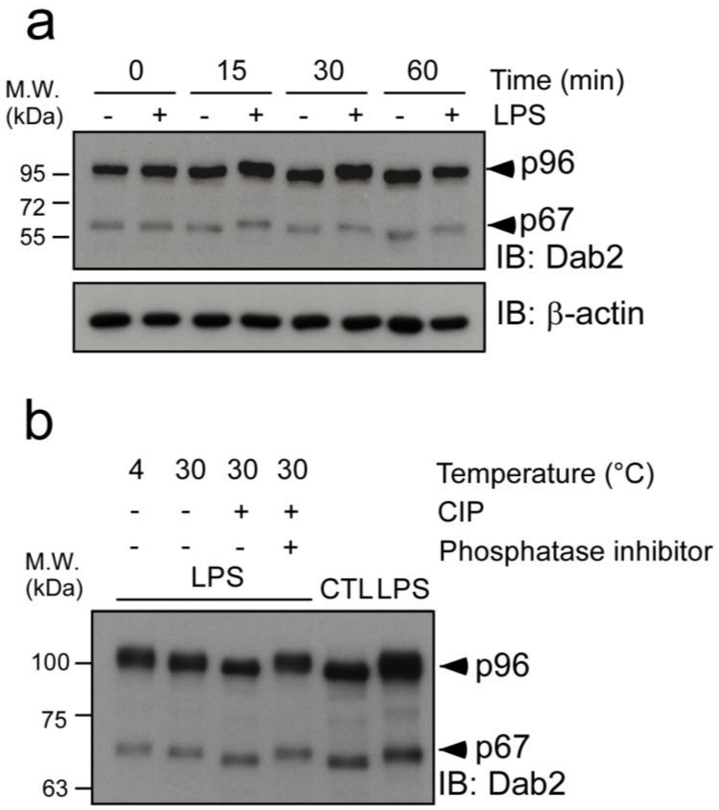

Supplementary Fig. S1 Dab2 was phosphorylated upon LPS stimulation.

(a) The shLuc cells were treated with LPS (100 ng/ml) for the indicated time. The cell lysates were collected for Western blotting using the antibody against Dab2. The expression of β-actin was used as a control for equal protein loading. **(b)** Reversal of gel mobility shift by phosphatase treatment. Protein extracts from shLuc cells treated with LPS for 30 min were kept at 4℃ or 30℃ in the presence of calf intestinal phosphatase (CIP) with or without the addition of the phosphatase inhibitors.

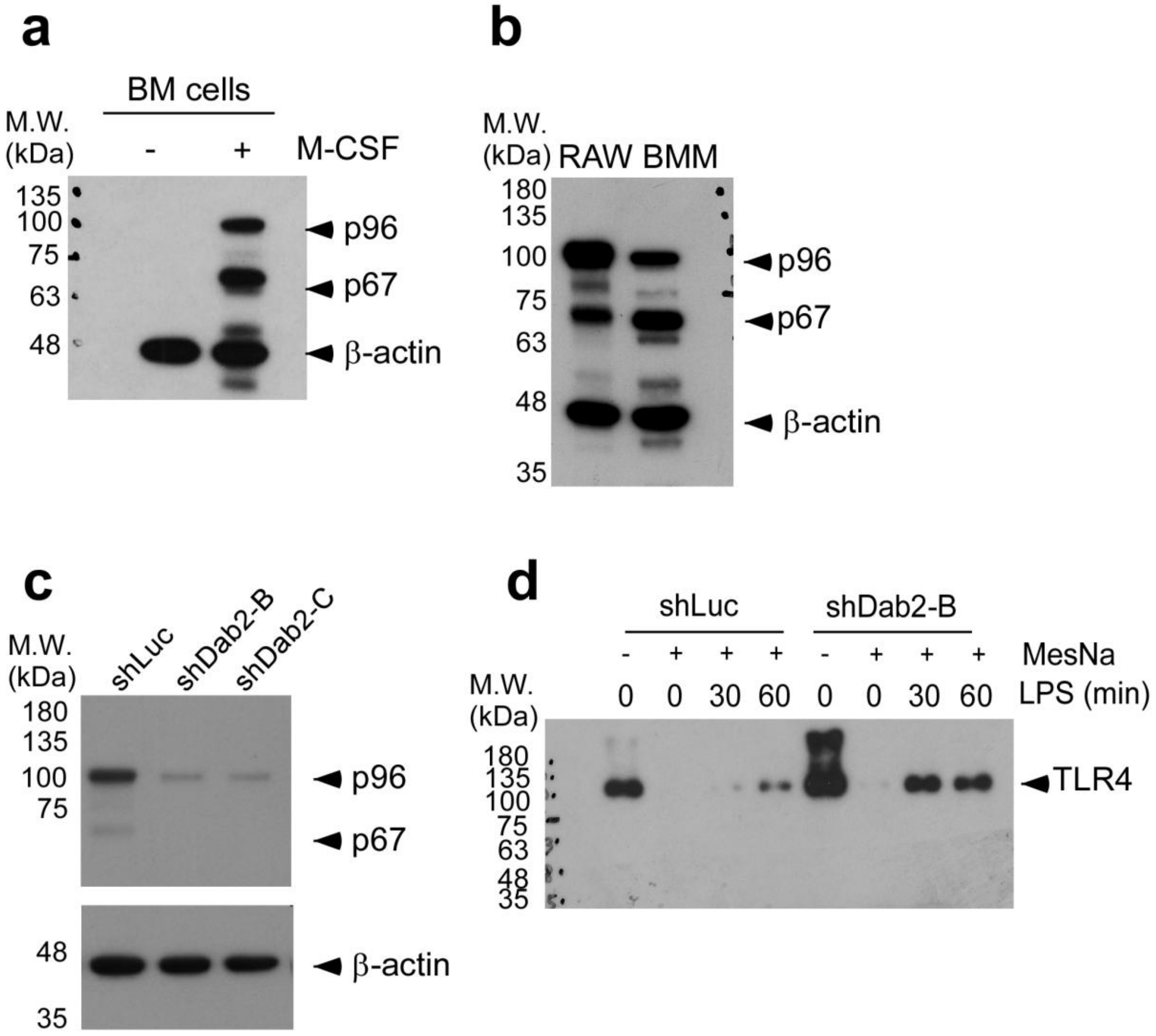

Supplementary Fig. S2 Full Western blots of Fig. 1b, Fig. 1c, Fig. 2a and Fig. 3d.

Fig. S2a: full Western blot of Fig. 1b.

Fig. S2b: full Western blot of Fig. 1c.

Fig. S2c: full Western blot of Fig. 2a.

Fig. S2d: full Western blot of Fig. 3d.

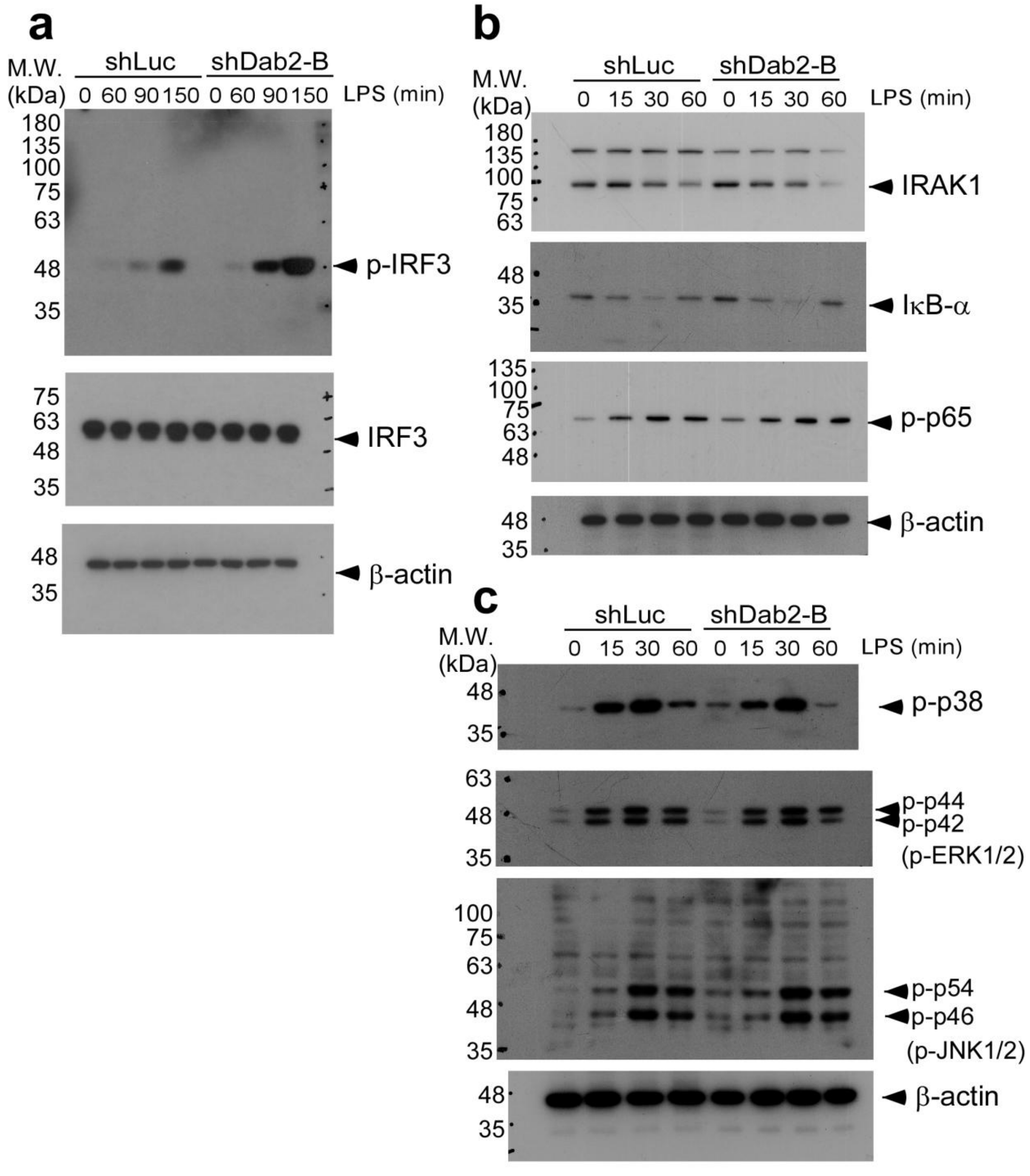

Supplementary Fig. S3 Full Western blots of Fig. 4a-c.

Fig. S3a: full Western blot of Fig. 4a .

Fig. S3b: full Western blot of Fig. 4b.

Fig. S3c: full Western blot of Fig. 4c.

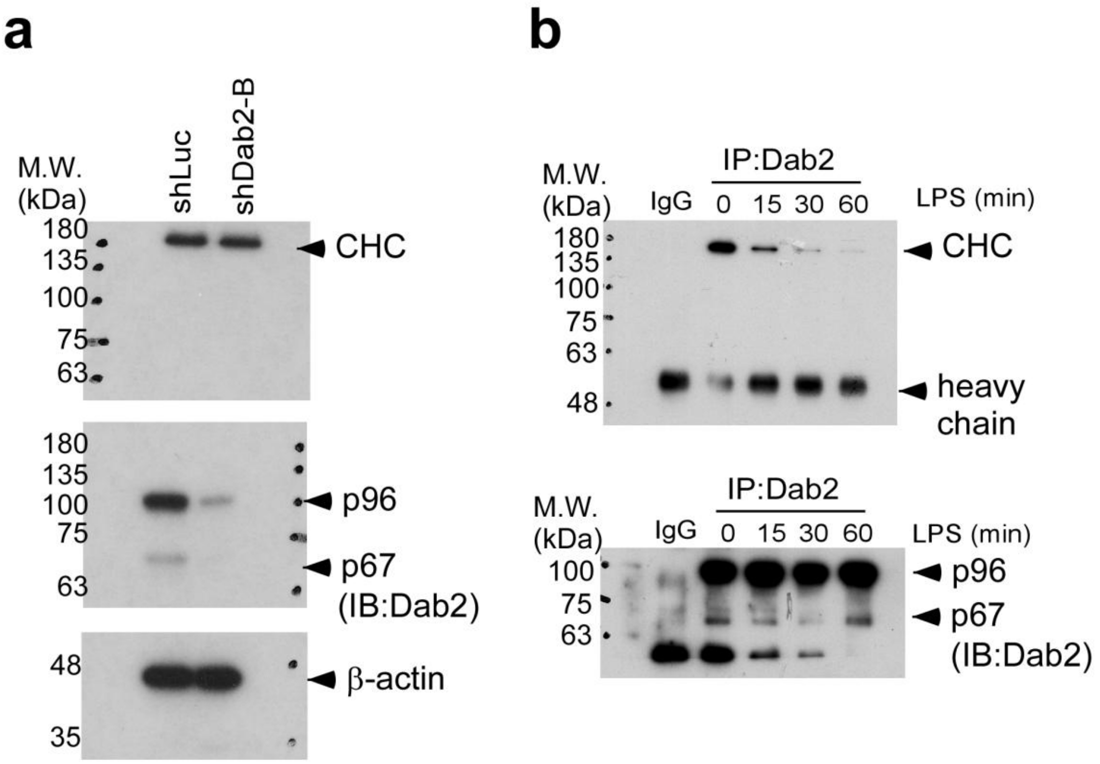

Supplementary Fig. S4 Full Western blots of Fig. 5b and Fig.5c

Fig. S4a: full Western blot of Fig. 5b.

Fig. S4b: full Western blot of Fig. 5c.

Table S1.-List of primer sets used in quantitative RT-PCR

No.	Gene	Primer sequences
**1**	**mTNF-α**	Fw: 5'-CTA TGG CCC AGA CCC TCA CAC TC -3'<br>Rv:5'-GCT GGC ACC ACT AGT TGG TTG TCT T 3'
**2**	**mIL-6**	Fw: 5'- CGT GGA AAT GAG AAA AGA GTT GTG-3'<br>Rv:5'-CCA GTT TGG TAG CAT CCA TCA TTT CT 3'
**3**	**mIFN-β**	Fw: 5'-GGC GGT TCC TTC GAG TGA CA-3'<br>Rv:5'-GATGG CAAAG GCAGT GTAAC TCTT3'
**4**	**mRANTES**	Fw: 5'-TGC CCT CAC CAT CAT CCT CAC T-3'<br>Rv:5'- GGC GGT TCC TTC GAG TGA CA-3'

Table S2. The differentially expressed genes between LPS-treated and untreated shLuc cells (LL/LC)

No.	GenBank accession no.	Gene Symbol	Log_2 Ratio	Fold Change
**Up-regulated genes**				
1	NM_009971.1	*Csf3*	6.64	100.00
2	NM_008361.3	*Il1b*	6.64	100.00
3	NM_009140.2	*Cxcl2*	6.64	100.00
4	NM_025404.3	*Arl4d*	6.64	99.72
5	NM_008392.1	*Irg1*	6.63	98.85
6	NM_007707.3	*Socs3*	5.52	45.79
7	NM_011198.3	*Ptgs2*	5.44	43.53
8	NM_013599.2	*Mmp9*	5.32	40.04
9	NM_177371.3	*Tnfsf15*	4.67	25.48
10	NM_007528.3	*Bcl6b*	4.56	23.56
11	NM_011333.3	*Ccl2*	4.20	18.44
12	NM_011610.3	*Tnfrsf1b*	4.17	17.98
13	NM_001159395.1	*Nfkbiz*	4.11	17.23
14	NM_001039701.3	*Il1rn*	4.05	16.56
15	NM_009344.3	*Phlda1*	4.02	16.23
16	NM_011113.3	*Plaur*	3.96	15.57
17	NM_007987.2	*Fas*	3.94	15.32
18	NM_010510.1	*Ifnb1*	3.74	13.34
19	NM_008871.2	*Serpine1*	3.73	13.27
20	NM_024406.2	*Fabp4*	3.71	13.09
21	NM_152804.2	*Plk2*	3.67	12.73
22	NM_011607.3	*Tnc*	3.67	12.73
23	NM_008321.2	*Id3*	3.67	12.70
24	NM_010484.2	*Slc6a4*	3.66	12.66
25	NM_013693.2	*Tnf*	3.62	12.30
26	NM_007836.1	*Gadd45a*	3.48	11.14
27	NM_009779.2	*C3ar1*	3.41	10.65
28	NM_011267.3	*Rgs16*	3.40	10.53
29	NM_001045526.2	*A430084P05Rik*	3.38	10.40
30	NM_009344.3	*Phlda1*	3.37	10.33

31	NM_023044.2	*Slc15a3*	3.33	10.06
32	NM_178890.3	*Abtb2*	3.31	9.91
33	NM_030720.1	*Gpr84*	3.30	9.83
34	NM_001025606.1	*Tmem171*	3.27	9.66
35	NM_023061.2	*Mcam*	3.24	9.47
36	NM_011057.3	*Pdgfb*	3.24	9.43
37	NM_030701.3	*Niacr1*	3.23	9.41
38	NM_019777.3	*Ikbke*	3.22	9.33
39	NM_009704.3	*Areg*	3.21	9.28
40	NM_001044384.1	*Timp1*	3.17	8.99
41	NM_019948.2	*Clec4e*	3.14	8.84
42	NM_145636.1	*Il27*	3.13	8.76
43	NM_025541.3	*Asf1a*	3.13	8.75
44	NM_133662.2	*Ier3*	3.12	8.69
45	NM_030701.3	*Niacr1*	3.12	8.67
46	NM_008230.5	*Hdc*	3.08	8.46
47	NM_009421.3	*Traf1*	3.04	8.21
48	NM_001177982.1	*Pde4b*	3.01	8.07
49	NM_026772.2	*Cdc42ep2*	2.97	7.84
50	NM_010235.2	*Fosl1*	2.93	7.61
51	NM_029478.3	*Vmp1*	2.92	7.58
52	NM_008655.1	*Gadd45b*	2.92	7.57
53	NM_009506.2	*Vegfc*	2.91	7.50
54	NM_008842.3	*Pim1*	2.86	7.25
55	NM_008655.1	*Gadd45b*	2.85	7.23
56	XR_140864.1	*5330417H12Rik*	2.83	7.13
57	NM_010807.4	*Marcksl1*	2.82	7.08
58	NM_009397.3	*Tnfaip3*	2.78	6.86
59	NM_172989.1	*Lpar1*	2.77	6.83
60	NM_011361.3	*Sgk1*	2.74	6.66
61	NM_001077189.1	*Fcgr2b*	2.73	6.64
62	NM_026644.2	*Agpat4*	2.71	6.56
63	NM_009396.2	*Tnfaip2*	2.70	6.49
64	NM_010187.2	*Fcgr2b*	2.70	6.48
65	NM_021274.2	*Cxcl10*	2.69	6.44
66	NM_153159.2	*Zc3h12a*	2.68	6.39

67	NM_009895.3	*Cish*	2.66	6.34
68	NM_025286.2	*Slc31a2*	2.63	6.19
69	NM_199241.2	*Sema6d*	2.63	6.19
70	NM_001146161.1	*Slc11a2*	2.63	6.17
71	NM_013807.2	*Plk3*	2.62	6.17
72	NM_207231.1	*Arl5c*	2.62	6.13
73	NM_007484.2	*Rhoc*	2.62	6.13
74	NM_001013365.2	*Osm*	2.61	6.11
75	NM_001048054.1	*Dusp16*	2.61	6.09
76	NM_008348.2	*Il10ra*	2.58	5.98
77	NM_029537.1	*Tmem98*	2.56	5.91
78	NM_174850.3	*Micall2*	2.56	5.88
79	NM_011110.4	*Pla2g5*	2.54	5.80
80	NM_009044.2	*Rel*	2.50	5.65
81	NM_026097.3	*Rffl*	2.48	5.57
82	NM_001005846.2	*Mcoln2*	2.48	5.56
83	NM_013867.2	*Bcar3*	2.47	5.53
84	NM_011338.2	*Ccl9*	2.46	5.50
85	NR_029806.1	*Mir221*	2.45	5.46
86	NM_176913.3	*Dpep2*	2.44	5.43
87	NM_021394.2	*Zbp1*	2.44	5.42
88	NM_008714.3	*Notch1*	2.44	5.42
89	NM_001081180.1	*Spink5*	2.43	5.40
90	NM_010208.4	*Fgr*	2.42	5.34
91	NM_010215.3	*Il4i1*	2.41	5.33
92	NM_015790.3	*Icosl*	2.39	5.25
93	NM_011426.3	*Siglec1*	2.38	5.19
94	NM_021384.4	*Rsad2*	2.35	5.11
95	NM_134102.4	*Pla1a*	2.33	5.05
96	NM_001191008.1	*Sstr5*	2.32	5.01
97	NM_019429.2	*Prss16*	2.32	4.98
98	NM_001048054.1	*Dusp16*	2.31	4.97
99	NM_010755.3	*Maff*	2.31	4.96
100	NM_145828.3	*Xylt2*	2.29	4.88
101	NM_133753.1	*Errfi1*	2.28	4.87

102	NM_001167680.1	*Rhbdf2*	2.28	4.86
103	NM_001039385.1	*Vgf*	2.27	4.83
104	NM_011756.4	*Zfp36*	2.25	4.75
105	NM_009743.4	*Bcl2l1*	2.25	4.75
106	NM_013612.2	*Slc11a1*	2.24	4.72
107	NM_017466.4	*Ccrl2*	2.23	4.71
108	NR_015566.2	*A330023F24Rik*	2.23	4.68
109	NM_028800.3	*Stk40*	2.22	4.66
110	NM_010090.2	*Dusp2*	2.22	4.65
111	NM_172833.2	*Malt1*	2.22	4.65
112	NM_177390.3	*Myo1d*	2.21	4.63
113	NM_009132.2	*Scin*	2.19	4.58
114	NM_025684.2	*Nepn*	2.19	4.57
115	NM_001163556.1	*Pou2f2*	2.19	4.56
116	NM_011577.1	*Tgfb1*	2.18	4.54
117	NM_010119.5	*Ehd1*	2.15	4.43
118	NM_007754.2	*Cpd*	2.14	4.42
119	NM_008416.3	*Junb*	2.13	4.39
120	NM_010577.3	*Itga5*	2.13	4.37
121	NM_019549.2	*Plek*	2.13	4.36
122	NM_011414.3	*Slpi*	2.11	4.32
123	NM_025638.2	*Gdpd1*	2.09	4.27
124	NM_026985.1	*1810033B17Rik*	2.07	4.20
125	NM_027455.2	*Qpct*	2.05	4.15
126	NM_010495.2	*Id1*	2.05	4.14
127	NM_001199305.1	*Atxn1*	2.04	4.11
128	NM_001033270.2	*Slc4a7*	2.04	4.10
129	NM_013652.2	*Ccl4*	2.03	4.09
130	NM_009612.2	*Acvrl1*	2.03	4.09
131	NM_018807.5	*Plagl2*	2.03	4.07
132	NM_011400.3	*Slc2a1*	2.02	4.06
133	NM_010403.2	*Hao1*	2.00	4.00
134	NM_001126047.1	*Sema4c*	2.00	3.99
135	NM_007413.4	*Adora2b*	1.99	3.97
136	NM_001099624.2	*Rapgef2*	1.99	3.96

137	NM_018764.2	*Pcdh7*	1.98	3.93
138	NM_009288.2	*Stk10*	1.96	3.90
139	NM_001163591.1	*Stx11*	1.96	3.88
140	NM_011408.1	*Slfn2*	1.94	3.84
141	NM_175437.3	*Pion*	1.94	3.83
142	NM_027871.1	*Arhgef3*	1.93	3.82
143	NM_001025395.2	*Src*	1.93	3.80
144	NM_001114088.1	*Pdlim7*	1.92	3.78
145	NM_008331.3	*Ifit1*	1.92	3.78
146	NM_011990.2	*Slc7a11*	1.90	3.74
147	NM_011227.1	*Rab20*	1.90	3.72
148	NM_013470.2	*Anxa3*	1.88	3.67
149	NM_018782.2	*Calcrl*	1.87	3.66
150	NM_001163591.1	*Stx11*	1.87	3.66
151	NM_175512.2	*Dhrs9*	1.86	3.63
152	NM_001040397.4	*Filip1l*	1.86	3.63
153	NM_001093766.1	*Myadm*	1.86	3.63
154	NM_013532.2	*Lilrb4*	1.85	3.61
155	NM_023380.2	*Samsn1*	1.84	3.59
156	NM_011410.2	*Slfn4*	1.83	3.56
157	NM_026929.4	*Chac1*	1.83	3.55
158	NM_001243050.1	*Atp6v0a1*	1.82	3.54
159	NM_183177.2	*Zfp811*	1.82	3.52
160	NM_028127.3	*Frmd6*	1.81	3.51
161	NM_026058.4	*Lass4*	1.80	3.49
162	NM_144797.3	*Metrnl*	1.80	3.48
163	NM_153119.2	*Plekho2*	1.80	3.47
164	NM_011777.2	*Zyx*	1.79	3.47
166	NM_201518.4	*Flrt2*	1.79	3.46
167	NM_027249.2	*Tlcd2*	1.78	3.43
168	NM_001042659.1	*Fzd5*	1.77	3.41
169	NM_183148.3	*Iffo2*	1.76	3.40
170	NM_019472.2	*Myo10*	1.76	3.40
171	NM_134133.2	*2010002N04Rik*	1.76	3.39
172	NM_029422.3	*Tm7sf4*	1.76	3.39

173	NM_015789.3	*Dkkl1*	1.76	3.38
174	NR_003508.1	*Mx2*	1.75	3.37
175	NM_019549.2	*Plek*	1.75	3.37
176	NM_080853.3	*Slc17a6*	1.74	3.35
177	NM_029021.1	*4833422F24Rik*	1.74	3.33
178	NM_001163565.1	*Ptpn5*	1.73	3.32
179	NM_001162921.1	*Zc3h12c*	1.72	3.28
180	NM_175645.3	*Xylt1*	1.71	3.28
181	NM_001135657.1	*Ptprj*	1.71	3.27
182	NM_011521.2	*Sdc4*	1.70	3.25
183	NM_011731.3	*Slc6a20b*	1.69	3.24
184	NM_008798.2	*Pdcd1*	1.69	3.23
185	XM_003084664.1	*Sh2d6*	1.69	3.22
186	NM_028696.3	*Obfc2a*	1.68	3.20
187	NM_176933.4	*Dusp4*	1.68	3.20
188	NM_008327.2	*Ifi202b*	1.66	3.17
189	NM_029847.4	*Arsk*	1.66	3.16
190	XR_141159.1	*5033430I15Rik*	1.65	3.15
191	NM_053109.3	*Clec2d*	1.65	3.13
192	NM_145953.2	*Cth*	1.64	3.13
193	NM_001040400.2	*Tet2*	1.64	3.12
194	NM_025821.2	*Carhsp1*	1.63	3.10
195	NM_008185.3	*Gstt1*	1.63	3.10
196	NM_145950.4	*Osgin2*	1.63	3.09
197	NM_008102.3	*Gch1*	1.62	3.08
198	NM_007656.4	*Cd82*	1.61	3.06
199	NM_001199305.1	*Atxn1*	1.61	3.05
200	NM_029083.2	*Ddit4*	1.61	3.05
201	NM_010696.3	*Lcp2*	1.61	3.05
202	NM_001077407.1	*Nrp2*	1.61	3.04
203	NM_207652.2	*Tsc22d1*	1.60	3.04
204	NM_175155.4	*Sash1*	1.60	3.04
205	NM_001205044.1	*Jarid2*	1.60	3.02
206	NM_001111060.1	*Cd59a*	1.59	3.01
207	NM_010029.2	*Ddx4*	1.58	2.98
208	NM_031195.2	*Msr1*	1.57	2.97

209	NM_172924.3	*C230081A13Rik*	1.57	2.97
210	NM_133833.2	*Dst*	1.57	2.97
211	NM_008416.3	*Junb*	1.57	2.96
212	NM_023516.5	*Hilpda*	1.56	2.96
213	NM_008518.2	*Ltb*	1.56	2.95
214	NM_001093766.1	*Myadm*	1.56	2.95
215	NM_001164220.1	*Trim13*	1.55	2.93
216	NM_021493.2	*Arhgap23*	1.55	2.93
217	NM_175093.2	*Trib3*	1.55	2.93
218	NM_080844.4	*Serpinc1*	1.55	2.93
219	NM_023516.5	*Hilpda*	1.54	2.91
220	NM_001142959.1	*Bcl2l15*	1.54	2.91
221	NM_001205053.1	*Jdp2*	1.54	2.90
222	NM_028807.3	*1200009I06Rik*	1.53	2.90
223	NM_172442.3	*Dtx4*	1.53	2.88
224	NM_181734.3	*Ttpal*	1.53	2.88
225	NM_015747.2	*Slc20a1*	1.52	2.87
226	NM_001190449.1	*Ddah2*	1.52	2.87
227	NM_172145.3	*Fam176b*	1.51	2.85
228	NM_025821.2	*Carhsp1*	1.51	2.85
229	NM_015783.3	*Isg15*	1.51	2.85
230	NM_029219.1	*Rnf19b*	1.51	2.85
231	NM_001113553.1	*Irak2*	1.50	2.83
232	NM_008795.2	*Cdk18*	1.49	2.82
233	NM_015766.2	*Ebi3*	1.49	2.81
234	NM_145456.2	*Zswim6*	1.49	2.80
235	NM_175092.3	*Rhof*	1.49	2.80
236	NM_201361.2	*Fam82a1*	1.48	2.79
237	NR_027888.1	*Sqrdl*	1.46	2.76
238	NM_145456.2	*Zswim6*	1.45	2.73
239	NM_007913.5	*Egr1*	1.45	2.73
**Down-regulated genes**				
1	NM_001252506.1	*St6gal1*	-4.08	16.94
2	NM_024124.3	*Hdac9*	-3.11	8.63
3	NM_145611.4	*Kank2*	-2.86	7.24
4	NM_198024.1	*Ranbp3l*	-2.82	7.08

5	NM_177000.3	*C130050O18Rik*	-2.73	6.61
6	NM_001081079.1	*Ogfrl1*	-2.65	6.28
7	NM_007426.3	*Angpt2*	-2.61	6.10
8	NM_001004156.2	*Plekhg5*	-2.55	5.87
9	NM_001081656.2	*Neurl1b*	-2.53	5.77
10	NM_198024.1	*Ranbp3l*	-2.49	5.61
11	NM_025768.2	*Grtp1*	-2.49	5.60
12	NM_020581.2	*Angptl4*	-2.47	5.52
13	NM_008278.2	*Hpgd*	-2.38	5.20
14	NM_008278.2	*Hpgd*	-2.37	5.17
15	NM_013495.2	*Cpt1a*	-2.37	5.16
16	NM_009025.2	*Rasa3*	-2.36	5.15
17	NM_001163687.1	*Naaa*	-2.31	4.96
18	NM_029116.2	*Kbtbd11*	-2.29	4.87
19	NM_001114312.1	*4930506M07Rik*	-2.27	4.81
20	NM_028340.1	*Susd3*	-2.25	4.77
21	NM_053261.2	*Impa2*	-2.22	4.66
22	NM_001170643.1	*Rnf144b*	-2.20	4.58
23	NM_028639.3	*Ttc7*	-2.19	4.55
24	NM_009846.2	*Cd24a*	-2.11	4.31
25	NM_027763.1	*Treml1*	-2.10	4.28
26	NM_134189.2	*Galnt10*	-2.05	4.14
27	NM_028039.2	*Esco2*	-2.05	4.14
28	NM_001195006.1	*Ndrg4*	-2.04	4.11
29	NM_001130186.1	*Phf17*	-2.02	4.05
30	NM_001080158.1	*Cenpm*	-2.01	4.02
31	NM_178667.4	*Tfdp2*	-2.01	4.02
32	NM_001085492.1	*Rere*	-2.01	4.02
33	NM_030198.3	*Gins3*	-1.99	3.96
34	NM_001081098.1	*Zfp362*	-1.97	3.92
35	XR_106143.1	*G430095P16Rik*	-1.96	3.89
36	NM_009154.2	*Sema5a*	-1.95	3.85
37	XM_003689164.1	*LOC100504500*	-1.95	3.85
38	NM_172838.3	*Slc16a12*	-1.93	3.81
39	NM_198305.2	*Klhl17*	-1.93	3.80
40	NM_145509.2	*5430435G22Rik*	-1.92	3.77

41	NM_001177867.1	*Sgol2*	-1.91	3.76
42	NM_020567.2	*Gmnn*	-1.91	3.75
43	NM_133746.5	*Calhm2*	-1.90	3.73
44	NM_024264.4	*Cyp27a1*	-1.90	3.73
45	NM_178699.4	*B930041F14Rik*	-1.89	3.70
46	NM_173047.3	*Cbr3*	-1.88	3.68
47	NM_009001.6	*Rab3a*	-1.88	3.67
48	NM_001159538.1	*Fgd2*	-1.88	3.67
49	NM_023326.2	*Bmyc*	-1.87	3.66
50	NM_178283.3	*Asb13*	-1.81	3.52
51	NM_198861.1	*BC046404*	-1.81	3.51
52	NM_001013368.5	*E2f8*	-1.80	3.49
53	NM_009387.2	*Tk1*	-1.80	3.48
54	NM_001143777.1	*Fam13c*	-1.78	3.45
55	NM_145462.2	*Haus4*	-1.74	3.34
56	NM_001146180.1	*Mtss1*	-1.73	3.31
57	NM_026003.2	*Smarca2*	-1.73	3.31
58	NM_146040.1	*Cdca7l*	-1.73	3.31
59	NM_023431.5	*Mum1*	-1.72	3.31
60	NM_028576.2	*1700106N22Rik*	-1.72	3.30
61	NM_001167592.2	*Gm17365*	-1.72	3.30
62	NM_172734.3	*Stk38l*	-1.71	3.27
63	NM_018822.3	*Sgsh*	-1.71	3.27
64	NM_008618.3	*Mdh1*	-1.70	3.25
65	NM_172756.2	*Ankle1*	-1.70	3.25
66	NM_019976.3	*Psrc1*	-1.69	3.23
67	NM_009593.2	*Abcg1*	-1.69	3.22
68	NR_040293.1	*2310040G24Rik*	-1.67	3.18
69	NM_011459.4	*Serpinb8*	-1.67	3.18
70	NM_026038.2	*2810055F11Rik*	-1.66	3.17
71	NM_023850.2	*Chst1*	-1.66	3.15
72	NM_001168492.1	*Pdcd4*	-1.66	3.15
73	NM_001163761.1	*6430548M08Rik*	-1.65	3.13
74	NM_011243.1	*Rarb*	-1.64	3.12
75	NM_001145832.1	*Kifc3*	-1.64	3.11
76	NM_009183.2	*St8sia4*	-1.64	3.11

77	NM_009079.2	*Rpl22*	-1.63	3.10
78	NM_010255.3	*Gamt*	-1.63	3.10
79	NM_146208.2	*Neil3*	-1.63	3.10
80	NM_017391.3	*Slc5a3*	-1.61	3.06
81	NM_008628.2	*Msh2*	-1.61	3.05
82	NM_153571.2	*Hscb*	-1.61	3.05
83	NM_009806.2	*Cask*	-1.60	3.04
84	NM_134042.2	*Aldh6a1*	-1.59	3.02
85	NM_009029.2	*Rb1*	-1.58	3.00
86	NM_145402.3	*Tmem51*	-1.58	2.99
87	NM_172560.3	*Cntrob*	-1.57	2.98
88	NM_197990.3	*1700025G04Rik*	-1.57	2.98
89	NM_026720.2	*Ankrd13d*	-1.57	2.97
90	NM_008021.4	*Foxm1*	-1.57	2.97
91	NM_001242424.1	*Fam105a*	-1.57	2.97
92	NM_027543.3	*Gpr173*	-1.57	2.96
93	NM_028149.1	*Fbxl20*	-1.57	2.96
94	NM_013769.2	*Tjp3*	-1.56	2.95
95	NM_001081241.2	*Fam65a*	-1.55	2.94
96	NR_027827.1	*A030009H04Rik*	-1.55	2.93
97	NM_027480.3	*Ankrd24*	-1.55	2.92
98	NM_008317.4	*Hyal1*	-1.54	2.91
99	NM_001013377.2	*E130306D19Rik*	-1.54	2.91
100	NM_001037134.1	*Ccne2*	-1.54	2.91
101	NM_023733.3	*Crot*	-1.53	2.88
102	NM_027715.1	*Otud1*	-1.52	2.87
103	NM_001163764.1	*Tcf19*	-1.52	2.86
104	NM_013627.5	*Pax6*	-1.51	2.85
105	NM_201239.3	*Rnase4*	-1.51	2.85
106	NM_001164101.1	*Add3*	-1.51	2.85
107	NM_011391.1	*Slc16a7*	-1.51	2.84
108	NM_001042653.1	*Oip5*	-1.51	2.84
109	NM_176837.2	*Arhgap18*	-1.51	2.84
110	NM_029766.2	*Dtl*	-1.50	2.83
111	NM_024459.2	*Ppp3r1*	-1.50	2.82
112	NM_001004164.2	*Gnptab*	-1.50	2.82

113	NM_001177544.1	*Hist1h2ao*	-1.49	2.80
114	NM_026662.4	*Prps2*	-1.48	2.80
115	NM_008845.4	*Pip4k2a*	-1.48	2.79
116	NM_146073.3	*Zdhhc14*	-1.48	2.79
117	NM_030087.2	*Ndufv3*	-1.47	2.78
118	NM_031182.2	*Tfap4*	-1.47	2.78
119	NM_001163766.1	*Wdr90*	-1.47	2.76
120	NR_033402.1	*Tyms*	-1.46	2.75
121	NM_007525.3	*Bard1*	-1.46	2.74
122	NM_178061.5	*Mob3b*	-1.46	2.74
123	NM_026321.4	*Fam174a*	-1.45	2.74
124	NM_139145.4	*Hlcs*	-1.45	2.74
125	NM_025995.2	*Fbxo5*	-1.45	2.74
126	NM_026975.2	*Bola1*	-1.45	2.74
127	NM_177124.4	*Tnrc6b*	-1.45	2.74

Table S3. The differentially expressed genes between LPS-treated and untreated shDab2-B cells (DL/DC)

No.	GenBank accession no.	Gene Symbol	Log_2 Ratio	Fold Change
**Up-regulated genes**				
1	NM_009971.1	*Csf3*	8.53	369.74
2	NM_008361.3	*Il1b*	7.7	207.62
3	NM_008392.1	*Irg1*	6.23	74.94
4	NM_025404.3	*Arl4d*	6.19	73.18
5	NM_007707.3	*Socs3*	6.03	65.5
6	NM_009140.2	*Cxcl2*	5.79	55.22
7	NM_177371.3	*Tnfsf15*	5.77	54.76
8	NM_011198.3	*Ptgs2*	5.15	35.42
9	NM_008230.5	*Hdc*	4.99	31.72
10	NM_013599.2	*Mmp9*	4.91	30.02
11	NM_011610.3	*Tnfrsf1b*	4.84	28.67
12	NM_001044384.1	*Timp1*	4.81	28.11
13	NM_008321.2	*Id3*	4.64	24.88
14	NM_001039701.3	*Il1rn*	4.52	23
15	NM_007836.1	*Gadd45a*	4.32	20.03
16	NM_017466.4	*Ccrl2*	4.28	19.47
17	NM_021384.4	*Rsad2*	4.24	18.9
18	NM_011113.3	*Plaur*	4.2	18.33
19	NM_010259.2	*Gbp1*	4.18	18.12
20	NM_009396.2	*Tnfaip2*	4.16	17.9
21	NR_033554.1	*D330041H03Rik*	4.11	17.3
22	NM_009895.3	*Cish*	4.03	16.33
23	NM_001077189.1	*Fcgr2b*	4.02	16.25
24	NM_013693.2	*Tnf*	4.01	16.07
25	NM_021394.2	*Zbp1*	4	15.99
26	NM_008655.1	*Gadd45b*	3.87	14.59
27	NM_001159395.1	*Nfkbiz*	3.83	14.21
28	NM_010187.2	*Fcgr2b*	3.82	14.13
29	NM_008655.1	*Gadd45b*	3.8	13.91
30	NM_030701.3	*Niacr1*	3.76	13.57
31	NM_023044.2	*Slc15a3*	3.76	13.53

32	NM_008331.3	*Ifit1*	3.74	13.39
33	NM_174850.3	*Micall2*	3.74	13.32
34	NM_030701.3	*Niacr1*	3.73	13.26
35	NM_021274.2	*Cxcl10*	3.67	12.71
36	NM_007987.2	*Fas*	3.66	12.65
37	NM_010484.2	*Slc6a4*	3.59	12.07
38	NM_015790.3	*Icosl*	3.5	11.34
39	NM_025541.3	*Asf1a*	3.47	11.11
40	NM_011333.3	*Ccl2*	3.47	11.09
41	NM_145636.1	*Il27*	3.42	10.68
42	NM_152804.2	*Plk2*	3.42	10.67
43	NM_053108.4	*Glrx*	3.36	10.28
44	NM_019777.3	*Ikbke*	3.32	10.01
45	NM_008842.3	*Pim1*	3.32	10
46	NM_134102.4	*Pla1a*	3.32	9.96
47	NM_207231.1	*Arl5c*	3.31	9.91
48	NM_010846.1	*Mx1*	3.3	9.84
49	NM_009344.3	*Phlda1*	3.29	9.76
50	NM_009344.3	*Phlda1*	3.28	9.69
51	NM_010260.1	*Gbp2*	3.26	9.59
52	NM_026929.4	*Chac1*	3.26	9.59
53	NM_030720.1	*Gpr84*	3.26	9.58
54	NM_009397.3	*Tnfaip3*	3.24	9.47
55	NM_011607.3	*Tnc*	3.19	9.1
56	NM_153159.2	*Zc3h12a*	3.16	8.91
57	NM_001013365.2	*Osm*	3.15	8.85
58	NM_133662.2	*Ier3*	3.13	8.77
59	NM_172989.1	*Lpar1*	3.13	8.74
60	NM_019948.2	*Clec4e*	3.12	8.69
61	NM_001177982.1	*Pde4b*	3.12	8.67
62	NM_001113527.1	*Isg20*	3.11	8.66
63	NM_008871.2	*Serpine1*	3.11	8.66
64	NM_013807.2	*Plk3*	3.09	8.54
65	NM_178890.3	*Abtb2*	3.09	8.51
66	NM_008329.2	*Ifi204*	3.08	8.48
67	NM_009421.3	*Traf1*	3.08	8.48
68	NM_172833.2	*Malt1*	3.02	8.12

69	NM_013653.3	*Ccl5*	3.01	8.08
70	NM_009704.3	*Areg*	2.99	7.94
71	NM_175093.2	*Trib3*	2.99	7.92
72	NM_026985.1	*1810033B17Rik*	2.97	7.84
73	NM_010807.4	*Marcksl1*	2.96	7.8
74	NM_001005846.2	*Mcoln2*	2.96	7.77
75	NM_010090.2	*Dusp2*	2.89	7.39
76	NM_001048054.1	*Dusp16*	2.85	7.21
77	NM_001025606.1	*Tmem171*	2.82	7.08
78	NM_010577.3	*Itga5*	2.82	7.06
79	NM_001005858.3	*I830012O16Rik*	2.82	7.04
80	NM_007528.3	*Bcl6b*	2.78	6.85
81	NM_018734.3	*Gbp3*	2.75	6.75
82	NM_011756.4	*Zfp36*	2.74	6.66
83	NM_015783.3	*Isg15*	2.72	6.58
84	NM_008332.3	*Ifit2*	2.71	6.53
85	NM_001163591.1	*Stx11*	2.71	6.52
86	NM_026097.3	*Rffl*	2.68	6.42
87	NM_027871.1	*Arhgef3*	2.67	6.35
88	NM_011267.3	*Rgs16*	2.64	6.23
89	NM_011361.3	*Sgk1*	2.64	6.23
90	NM_025286.2	*Slc31a2*	2.64	6.22
91	NM_011227.1	*Rab20*	2.61	6.09
92	NM_009506.2	*Vegfc*	2.6	6.06
93	NM_009044.2	*Rel*	2.6	6.05
94	NM_010235.2	*Fosl1*	2.59	6.02
95	NM_011426.3	*Siglec1*	2.58	5.96
96	NM_008102.3	*Gch1*	2.57	5.95
97	NM_001025395.2	*Src*	2.57	5.94
98	NM_001048054.1	*Dusp16*	2.56	5.9
99	NM_011057.3	*Pdgfb*	2.54	5.82
100	NM_001002927.2	*Penk*	2.54	5.82
101	NM_001045526.2	*A430084P05Rik*	2.53	5.77
102	NM_026772.2	*Cdc42ep2*	2.5	5.65
103	NM_008714.3	*Notch1*	2.46	5.5
104	NM_001146161.1	*Slc11a2*	2.45	5.47
105	NM_029537.1	*Tmem98*	2.45	5.47

106	NM_007754.2	*Cpd*	2.45	5.46
107	NM_008416.3	*Junb*	2.42	5.37
108	NM_001164477.1	*Ifih1*	2.42	5.36
109	NM_001040397.4	*Filip1l*	2.41	5.31
110	NM_028800.3	*Stk40*	2.4	5.28
111	NM_134133.2	*2010002N04Rik*	2.39	5.25
112	NM_007498.3	*Atf3*	2.38	5.22
113	NM_133753.1	*Errfi1*	2.37	5.18
114	NM_011410.2	*Slfn4*	2.36	5.13
115	NM_001163591.1	*Stx11*	2.34	5.08
116	NM_001191008.1	*Sstr5*	2.34	5.07
117	NM_013612.2	*Slc11a1*	2.34	5.06
118	NM_009779.2	*C3ar1*	2.33	5.02
119	NM_011408.1	*Slfn2*	2.32	5
120	NM_008654.2	*Ppp1r15a*	2.32	4.99
121	NM_001164220.1	*Trim13*	2.31	4.97
122	NM_008348.2	*Il10ra*	2.3	4.93
123	NM_011854.2	*Oasl2*	2.28	4.84
124	NM_010119.5	*Ehd1*	2.26	4.78
125	NR_027888.1	*Sqrdl*	2.25	4.77
126	NM_010208.4	*Fgr*	2.25	4.76
127	XM_003084664.1	*Sh2d6*	2.25	4.76
128	NR_029565.1	*Mir155*	2.23	4.7
129	NM_001243039.1	*Gvin1*	2.23	4.68
130	NM_020557.4	*Cmpk2*	2.22	4.65
131	NM_183201.4	*Slfn5*	2.21	4.64
132	NM_026644.2	*Agpat4*	2.21	4.61
133	NM_001081180.1	*Spink5*	2.2	4.58
134	NM_001093766.1	*Myadm*	2.17	4.5
135	NM_010755.3	*Maff*	2.16	4.46
136	NM_028127.3	*Frmd6*	2.15	4.43
137	NM_008416.3	*Junb*	2.14	4.42
138	NM_013470.2	*Anxa3*	2.13	4.38
139	NM_023061.2	*Mcam*	2.13	4.38
140	NM_001142706.1	*Cfb*	2.13	4.37
141	NM_176913.3	*Dpep2*	2.11	4.31
142	NM_009132.2	*Scin*	2.11	4.31

143	NM_001082552.1	*Trim21*	2.09	4.26
144	NM_008132.2	*Glrp1*	2.08	4.22
145	NM_027494.3	*Zcchc8*	2.05	4.15
146	NM_001159301.1	*Lgals9*	2.05	4.15
147	NM_001145835.1	*Ralgds*	2.05	4.13
148	NM_001163565.1	*Ptpn5*	2.05	4.13
149	NM_001167680.1	*Rhbdf2*	2.03	4.07
150	NM_145953.2	*Cth*	2.02	4.06
151	NM_145209.3	*Oasl1*	2.02	4.05
152	NM_015789.3	*Dkkl1*	2.01	4.03
153	NM_028807.3	*1200009I06Rik*	2.01	4.02
154	NM_028967.1	*Batf2*	1.99	3.98
155	NM_008327.2	*Ifi202b*	1.97	3.91
156	NM_013532.2	*Lilrb4*	1.97	3.91
157	NM_011338.2	*Ccl9*	1.97	3.91
158	XR_001627.2	*Gm8995*	1.96	3.88
159	NM_018807.5	*Plagl2*	1.95	3.86
160	NM_001045514.2	*Akna*	1.94	3.85
161	NM_011521.2	*Sdc4*	1.94	3.83
162	NM_207652.2	*Tsc22d1*	1.94	3.83
163	NM_019549.2	*Plek*	1.93	3.82
164	NM_008330.1	*Ifi47*	1.93	3.81
165	NM_015747.2	*Slc20a1*	1.93	3.8
166	NM_009288.2	*Stk10*	1.92	3.79
167	NM_008630.2	*Mt2*	1.91	3.77
168	NM_145828.3	*Xylt2*	1.91	3.76
169	NM_001004174.1	*AA467197*	1.91	3.76
170	NM_001099624.2	*Rapgef2*	1.91	3.75
171	NM_013673.3	*Sp100*	1.9	3.73
172	NR_003508.1	*Mx2*	1.9	3.73
173	NM_001164220.1	*Trim13*	1.9	3.73
174	NM_011777.2	*Zyx*	1.89	3.71
175	NM_177371.3	*Tnfsf15*	1.88	3.69
176	NM_001093766.1	*Myadm*	1.88	3.68
177	NM_013867.2	*Bcar3*	1.88	3.68
178	NM_028679.3	*Irak3*	1.86	3.63
179	NM_011400.3	*Slc2a1*	1.85	3.61

180	NM_025638.2	*Gdpd1*	1.85	3.6
181	NM_007413.4	*Adora2b*	1.85	3.6
182	NR_029806.1	*Mir221*	1.85	3.59
183	NM_175437.3	*Pion*	1.85	3.59
184	NM_023516.5	*Hilpda*	1.84	3.58
185	NM_011990.2	*Slc7a11*	1.83	3.57
186	NM_023380.2	*Samsn1*	1.83	3.56
187	NM_008102.3	*Gch1*	1.83	3.55
188	NM_007484.2	*Rhoc*	1.82	3.53
189	NM_030684.3	*Trim34a*	1.82	3.52
190	NM_023516.5	*Hilpda*	1.79	3.45
191	NM_018782.2	*Calcrl*	1.78	3.45
192	NM_011110.4	*Pla2g5*	1.78	3.44
193	NM_172603.3	*Phf1*	1.78	3.43
194	NM_011577.1	*Tgfb1*	1.78	3.43
195	NM_013652.2	*Ccl4*	1.78	3.43
196	NM_001199276.1	*Tnip1*	1.77	3.41
197	NM_009743.4	*Bcl2l1*	1.77	3.41
198	XM_003086779.1	*LOC100504934*	1.77	3.4
199	NM_001039530.3	*Parp14*	1.76	3.4
200	NM_001205044.1	*Jarid2*	1.76	3.38
201	NM_029083.2	*Ddit4*	1.76	3.38
202	NM_176933.4	*Dusp4*	1.76	3.38
203	NM_019738.1	*Nupr1*	1.75	3.37
204	NM_007729.2	*Col11a1*	1.75	3.36
205	NM_001199305.1	*Atxn1*	1.75	3.36
206	NM_001126047.1	*Sema4c*	1.75	3.36
207	NM_001172117.1	*Hck*	1.74	3.34
208	NM_001135657.1	*Ptprj*	1.72	3.3
209	NM_177794.3	*Tmem26*	1.72	3.3
210	NM_010499.4	*Ier2*	1.71	3.28
211	NM_032000.2	*Trps1*	1.71	3.27
212	NM_028186.4	*Nkd2*	1.71	3.27
213	NM_153074.3	*Lrrc25*	1.7	3.25
214	NM_144797.3	*Metrnl*	1.7	3.25
215	NM_001162883.1	*Apol9*	1.7	3.25
216	NM_001048177.1	*Jak2*	1.7	3.24

217	NM_001199733.1	*Daxx*	1.7	3.24
218	NM_018738.4	*Igtp*	1.69	3.23
219	NM_053244.5	*Kiss1r*	1.68	3.21
220	NM_007392.2	*Acta2*	1.66	3.16
221	NM_019549.2	*Plek*	1.65	3.14
222	NM_183148.3	*Iffo2*	1.64	3.12
223	NM_010442.2	*Hmox1*	1.64	3.11
224	NM_138953.2	*Ell2*	1.64	3.11
225	NM_172145.3	*Fam176b*	1.63	3.11
226	NM_008326.1	*Irgm1*	1.63	3.1
227	NM_011018.2	*Sqstm1*	1.63	3.1
228	NR_015466.1	*7530420F21Rik*	1.63	3.1
229	NM_013671.3	*Sod2*	1.62	3.07
230	NM_199241.2,	*Sema6d*	1.61	3.05
231	NM_011731.3	*Slc6a20b*	1.58	2.99
232	NM_010908.4	*Nfkbib*	1.58	2.98
233	XR_140714.1	*Gm9640*	1.57	2.98
234	NM_010720.3	*Lipg*	1.55	2.94
235	NM_172442.3	*Dtx4*	1.55	2.92
236	NM_021334.2	*Itgax*	1.54	2.91
237	NM_010495.2	*Id1*	1.54	2.91
238	NM_001037298.1	*Fam38a*	1.53	2.89
239	NM_019440.3	*Irgm2*	1.52	2.87
240	NM_010499.4	*Ier2*	1.52	2.86
241	NM_026835.2	*Ms4a6d*	1.52	2.86
242	NM_001177370.1	*Nfkb2*	1.51	2.85
243	NM_001160379.1	*Fam46a*	1.5	2.83
244	NM_029478.3	*Vmp1*	1.5	2.83
245	NR_015566.2	*A330023F24Rik*	1.5	2.82
246	NM_001160379.1	*Fam46a*	1.5	2.82
247	NM_022331.1	*Herpud1*	1.5	2.82
248	NM_001162921.1	*Zc3h12c*	1.49	2.82
249	NM_175512.2	*Dhrs9*	1.49	2.82
250	XM_001477846.2	*Rnf213*	1.49	2.81
251	NM_153543.2	*Aldh1l2*	1.49	2.81
252	NM_017373.3	*Nfil3*	1.49	2.8
253	NM_145950.4	*Osgin2*	1.48	2.8

254	NM_001252601.1	*Irf7*	1.48	2.79
255	NM_025821.2	*Carhsp1*	1.48	2.79
256	NM_001040400.2	*Tet2*	1.48	2.79
257	NM_009627.1	*Adm*	1.48	2.79
258	NR_028478.1	*Snora75*	1.47	2.78
259	NM_001163556.1	*Pou2f2*	1.46	2.76
260	NM_011212.3	*Ptpre*	1.46	2.75
261	NM_001163470.1	*Trafd1*	1.46	2.75
262	NM_080844.4	*Serpinc1*	1.45	2.74
263	NM_183162.2	*BC006779*	1.45	2.74
264	NM_172684.2	*Rsbn1*	1.45	2.73
265	NM_018861.3	*Slc1a4*	1.45	2.73
266	NM_007679.4	*Cebpd*	1.45	2.72
**Down-regulated genes**				
1	NM_001252506.1	*St6gal1*	-6.05	66.27
2	NM_029116.2	*Kbtbd11*	-4.47	22.17
3	NM_007642.4	*Cd28*	-4.39	20.9
4	NM_001081656.2	*Neurl1b*	-4.23	18.79
5	NM_025768.2	*Grtp1*	-4.13	17.54
6	NM_022886.2	*Scel*	-4.12	17.37
7	NM_144879.2	*Vash2*	-4.07	16.76
8	NM_020581.2	*Angptl4*	-3.96	15.6
9	NM_027543.3	*Gpr173*	-3.95	15.48
10	NM_007426.3	*Angpt2*	-3.9	14.92
11	NM_198024.1	*Ranbp3l*	-3.84	14.3
12	NM_145611.4	*Kank2*	-3.72	13.17
13	NM_024124.3	*Hdac9*	-3.71	13.05
14	NM_008278.2	*Hpgd*	-3.62	12.26
15	NM_198024.1	*Ranbp3l*	-3.58	11.96
16	NM_011459.4	*Serpinb8*	-3.56	11.77
17	NM_001167592.2	*Gm17365*	-3.5	11.32
18	NM_001004156.2	*Plekhg5*	-3.47	11.1
19	NM_023850.2	*Chst1*	-3.45	10.96
20	NM_001130186.1	*Phf17*	-3.39	10.51
21	NM_011027.2	*P2rx7*	-3.38	10.38
22	XM_003689164.1	*LOC100504500*	-3.37	10.31
23	NM_177000.3	*C130050O18Rik*	-3.34	10.13

24	NM_008278.2	*Hpgd*	-3.31	9.94
25	NM_017480.2	*Icos*	-3.31	9.89
26	NM_028340.	*Susd3*	-3.3	9.88
27	NM_001143777.1	*Fam13c*	-3.28	9.72
28	NM_001170643.1	*Rnf144b*	-3.25	9.54
29	NM_028039.2	*Esco2*	-3.24	9.46
30	NM_178667.4	*Tfdp2*	-3.19	9.12
31	NM_028149.1	*Fbxl20*	-3.16	8.97
32	NM_001163616.1	*1810011H11Rik*	-3.13	8.77
33	NM_009025.2	*Rasa3*	-3.09	8.54
34	NM_178699.4	*B930041F14Rik*	-3.08	8.48
35	NM_001195006.1	*Ndrg4*	-3.08	8.46
36	NM_001168492.1	*Pdcd4*	-3.08	8.46
37	NM_013627.5	*Pax6*	-3.08	8.45
38	NM_053261.2	*Impa2*	-3.06	8.34
39	NM_011243.1	*Rarb*	-3.06	8.33
40	NM_001039231.3	*Zfp951*	-3.05	8.3
41	NM_001204241.1	*Clec4a3*	-3.04	8.24
42	NM_001162425.1	*Efna1*	-3.04	8.2
43	NM_001081098.1	*Zfp362*	-3.03	8.16
44	NM_013495.2	*Cpt1a*	-3.02	8.11
45	NM_001168491.1	*Pdcd4*	-2.97	7.84
46	NM_133746.5	*Calhm2*	-2.97	7.81
47	NM_001081079.1	*Ogfrl1*	-2.96	7.78
48	NM_027763.1	*Treml1*	-2.95	7.74
49	NM_001199105.1	*Trp53inp1*	-2.94	7.67
50	NM_001114312.1	*4930506M07Rik*	-2.94	7.67
51	NM_001037134.1	*Ccne2*	-2.93	7.64
52	NM_025294.5	*Gm16515*	-2.92	7.55
53	NM_001190950.1	*Kcne3*	-2.9	7.46
54	NM_146208.2	*Neil3*	-2.9	7.46
55	NM_025995.2	*Fbxo5*	-2.88	7.38
56	NM_001013368.5	*E2f8*	-2.87	7.31
57	NM_030198.3	*Gins3*	-2.87	7.31
58	NM_018822.3	*Sgsh*	-2.87	7.3
59	NM_001013377.2	*E130306D19Rik*	-2.87	7.29
60	NM_145434.3	*Nr1d1*	-2.86	7.26

61	NM_009846.2	*Cd24a*	-2.85	7.22
62	NM_001163256.1	*Fblim1*	-2.85	7.2
63	NM_007930.4	*Enc1*	-2.84	7.18
64	NM_017391.3	*Slc5a3*	-2.83	7.12
65	NM_001145959.1	*Ndrg2*	-2.82	7.05
66	NM_134189.2	*Galnt10*	-2.82	7.05
67	NM_133898.4	*N4bp2l1*	-2.81	7.02
68	NM_172756.2	*Ankle1*	-2.8	6.98
69	NM_001130412.1	*Lpin1*	-2.8	6.95
70	XR_106143.1	*G430095P16Rik*	-2.79	6.93
71	NM_172598.3	*Wdhd1*	-2.79	6.89
72	NM_153388.4	*Lrfn4*	-2.78	6.86
73	NM_183031.2	*Gpr183*	-2.77	6.83
74	NM_145462.2	*Haus4*	-2.77	6.81
75	NM_028763.3	*Cbx6*	-2.76	6.78
76	NM_138313.3	*Bmf*	-2.75	6.72
77	NM_026038.2	*2810055F11Rik*	-2.75	6.71
78	NM_028995.3	*Nipal3*	-2.74	6.68
79	NM_001145832.1	*Kifc3*	-2.74	6.68
80	NM_146040.1	*Cdca7l*	-2.73	6.62
81	NM_001113283.1	*BC031353*	-2.71	6.56
82	NM_176837.2	*Arhgap18*	-2.71	6.55
83	NM_001170643.1	*Rnf144b*	-2.71	6.55
84	NM_009806.2	*Cask*	-2.7	6.52
85	NM_001163687.1	*Naaa*	-2.69	6.47
86	NM_001080158.1	*Cenpm*	-2.68	6.41
87	NM_010049.3	*Dhfr*	-2.67	6.38
88	NM_198861.1	*BC046404*	-2.67	6.37
89	NM_177603.3	*Frat2*	-2.66	6.32
90	NM_010412.3	*Hdac5*	-2.66	6.32
91	NM_001085410.1	*1110020G09Rik*	-2.66	6.31
92	NM_001005421.4	*Amica1*	-2.65	6.26
93	NM_178220.3	*Arrb1*	-2.64	6.23
94	NM_023794.2	*Etv5*	-2.64	6.23
95	NM_145402.3	*Tmem51*	-2.63	6.19
96	NM_197990.3	*1700025G04Rik*	-2.62	6.15
97	NM_001033306.1	*Shb*	-2.62	6.14

98	NM_177733.6	*E2f2*	-2.61	6.12
99	NM_009001.6	*Rab3a*	-2.61	6.12
100	NM_001164112.1	*Nfatc1*	-2.61	6.1
101	NM_020567.2	*Gmnn*	-2.61	6.09
102	NM_198305.2	*Klhl17*	-2.6	6.07
103	NM_009593.2	*Abcg1*	-2.6	6.04
104	NM_153571.2	*Hscb*	-2.59	6.02
105	NM_001114312.1	*4930506M07Rik*	-2.59	6.02
106	NM_023397.4	*Mdp1*	-2.58	5.98
107	NM_023326.2	*Bmyc*	-2.57	5.92
108	NM_001164101.1	*Add3*	-2.56	5.91
109	NM_024459.2	*Ppp3r1*	-2.56	5.9
110	NM_008628.2	*Msh2*	-2.56	5.89
111	NM_025464.2	*Tmem218*	-2.54	5.81
112	NM_173047.3	*Cbr3*	-2.53	5.77
113	NM_001085492.1	*Rere*	-2.53	5.77
114	NM_030198.3	*Gins3*	-2.52	5.72
115	NM_145425.3	*Wdpcp*	-2.51	5.71
116	NR_027827.1	*A030009H04Rik*	-2.5	5.67
117	NM_027973.3	*Mlf1ip*	-2.49	5.61
118	NM_009079.2	*Rpl22*	-2.48	5.59
119	NM_001242424.1	*Fam105a*	-2.47	5.53
120	NM_007525.3	*Bard1*	-2.46	5.5
121	NM_023733.3	*Crot*	-2.44	5.44
122	NM_146073.3	*Zdhhc14*	-2.44	5.44
123	NM_009154.2	*Sema5a*	-2.43	5.38
124	NM_172838.3	*Slc16a12*	-2.42	5.37
125	NM_181397.2	*Rftn1*	-2.42	5.35
126	NM_178609.4	*E2f7*	-2.42	5.34
127	NM_001115018.1	*Suv420h2*	-2.42	5.34
128	NM_001163766.1	*Wdr90*	-2.41	5.33
129	NM_029091.2	*Klc4*	-2.41	5.32
130	NM_028639.3	*Ttc7*	-2.41	5.3
131	NM_134126.3	*Ift140*	-2.4	5.29
132	NM_001163764.1	*Tcf19*	-2.39	5.24
133	NM_011391.1	*Slc16a7*	-2.39	5.24
134	NM_001033162.2	*1700012A16Rik*	-2.38	5.22

135	NR_033402.1	*Tyms*	-2.38	5.21
136	NM_183031.2	*Gpr183*	-2.38	5.2
137	NM_001013370.2,	*Sesn1*	-2.37	5.17
138	NM_009662.2	*Alox5*	-2.36	5.14
139	NM_176831.4	*Ppcdc*	-2.36	5.14
140	NM_023431.5	*Mum1*	-2.36	5.13
141	NM_153546.4	*Mboat1*	-2.35	5.11
142	NM_145823.2	*Pitpnc1*	-2.35	5.11
143	NM_001146176.1	*Max*	-2.35	5.08
144	NM_172560.3	*Cntrob*	-2.34	5.06
145	NM_028995.3	*Nipal3*	-2.34	5.05
146	NM_178283.3	*Asb13*	-2.32	5
147	NM_021476.4	*Cysltr1*	-2.32	4.99
148	NM_001159538.1	*Fgd2*	-2.31	4.96
149	NM_026410.3	*Cdca5*	-2.31	4.95
150	NM_146008.2	*Tcp11l2*	-2.3	4.93
151	NM_009828.2	*Ccna2*	-2.3	4.93
152	NM_025823.4	*Pcyox1*	-2.3	4.92
153	NM_134250.2	*Havcr2*	-2.29	4.9
154	NM_001163761.1	*6430548M08Rik*	-2.29	4.9
155	NM_001163518.1	*2410075B13Rik*	-2.29	4.9
156	NM_198019.2	*Cep78*	-2.29	4.89
157	NM_016681.3	*Chek2*	-2.27	4.84
158	NM_133762.3	*Ncapg2*	-2.27	4.83
159	NM_019976.3	*Psrc1*	-2.27	4.82
160	NM_026720.2	*Ankrd13d*	-2.27	4.81
161	NR_040293.1	*2310040G24Rik*	-2.26	4.8
162	NM_008737.2	*Nrp1*	-2.26	4.8
163	NM_139145.4	*Hlcs*	-2.26	4.8
164	NM_172310.2	*Tarsl2*	-2.26	4.8
165	NM_001081415.1	*Samd1*	-2.26	4.79
166	NM_009029.2	*Rb1*	-2.26	4.78
167	NM_025341.3	*Abhd6*	-2.26	4.78
168	NM_024264.4	*Cyp27a1*	-2.25	4.76
169	NM_026662.4	*Prps2*	-2.25	4.76
170	NM_001081241.2	*Fam65a*	-2.25	4.75
171	NM_178677.4	*Sec22c*	-2.25	4.75

172	NM_025341.3	*Abhd6*	-2.24	4.72
173	NM_028232.2	*Sgol1*	-2.23	4.7
174	NM_001146180.1	*Mtss1*	-2.23	4.69
175	NR_030721.1	*9130206I24Rik*	-2.23	4.69
176	NM_010255.3	*Gamt*	-2.23	4.68
177	NM_009427.2	*Tob1*	-2.21	4.63
178	NM_008021.4	*Foxm1*	-2.21	4.63
179	NM_028576.2	*1700106N22Rik*	-2.21	4.63
180	NM_029766.2	*Dtl*	-2.21	4.61
181	NM_024208.4	*Echdc3*	-2.2	4.61
182	NM_172260.3	*Cep68*	-2.2	4.61
183	NM_001001806.2	*Zfp36l2*	-2.2	4.6
184	NR_015585.1	*4933439C10Rik*	-2.19	4.58
185	NM_010830.2	*Msh6*	-2.19	4.58
186	NM_138956.3	*Rassf3*	-2.19	4.57
187	NM_007421.2	*Adssl1*	-2.19	4.57
188	NM_144526.3	*Fam64a*	-2.19	4.55
189	NM_177124.4	*Tnrc6b*	-2.18	4.53
190	NM_177473.3	*Tmem191c*	-2.18	4.53
191	NM_181039.2	*Lphn1*	-2.18	4.53
192	NM_175383.2	*B3gnt1*	-2.18	4.52
193	NM_019455.4	*Hpgds*	-2.17	4.51
194	NM_021886.1	*Cenph*	-2.17	4.5
195	NM_029482.1	*4930579G24Rik*	-2.17	4.5
196	NM_013926.1	*Cbx8*	-2.17	4.49
197	NM_013788.2	*Peg12*	-2.17	4.49
198	NM_201239.3	*Rnase4*	-2.16	4.47
199	NM_010620.1	*Kif15*	-2.15	4.44
200	NM_001170537.1	*Mef2c*	-2.15	4.43
201	NM_175449.4	*Fam26f*	-2.15	4.43
202	NM_007633.2	*Ccne1*	-2.15	4.43
203	NM_001033352.3	*Klhl21*	-2.15	4.43
204	NM_001170556.1	*Prkag2*	-2.14	4.42
205	NM_173733.3	*Suox*	-2.14	4.41
206	NM_009791.4	*Aspm*	-2.13	4.38
207	NM_013750.2	*Phlda3*	-2.13	4.37
208	NM_026967.4	*Rhebl1*	-2.12	4.35

209	NM_001177544.1	*Hist1h2ao*	-2.12	4.33
210	NM_001005420.1	*Ppp1r26*	-2.11	4.32
211	NM_026875.2	*Ypel3*	-2.11	4.31
212	NM_028543.3	*Zfp763*	-2.11	4.31
213	NM_175384.4	*Cdca2*	-2.11	4.3
214	NM_175554.4	*Clspn*	-2.1	4.3
215	NM_178607.4	*Rnf24*	-2.1	4.28
216	NM_177620.4	*Rin3*	-2.09	4.26
217	NM_010424.4	*Hfe*	-2.09	4.26
218	NM_008317.4	*Hyal1*	-2.08	4.24
219	NM_172734.3	*Stk38l*	-2.08	4.24
220	NM_144538.2	*Rab3il1*	-2.08	4.23
221	NM_008652.2	*Mybl2*	-2.08	4.23
222	NM_001033142.2	*Rnf166*	-2.07	4.21
223	NM_010188.5	*Fcgr3*	-2.07	4.21
224	NM_178694.3	*Zer1*	-2.07	4.19
225	NM_138719.4	*Gnb5*	-2.07	4.19
226	NM_031257.3	*Plekha2*	-2.06	4.18
227	NM_028083.4	*Chaf1b*	-2.06	4.18
228	NM_178214.3	*Hist2h2be*	-2.06	4.18
229	NM_134050.4	*Rab15*	-2.06	4.16
230	NM_134122.2	*Nrm*	-2.05	4.13
231	NM_026436.3	*Tmem86a*	-2.04	4.13
232	NM_030234.2	*Wdr76*	-2.04	4.11
233	NM_178162.2	*Agfg2*	-2.04	4.1
234	NM_026166.2	*Ikbip*	-2.04	4.1
235	NM_012012.4	*Exo1*	-2.03	4.1
236	NM_008832.2	*Phka1*	-2.03	4.1
237	NM_001191044.1	*Ddx43*	-2.03	4.09
238	NM_009387.2	*Tk1*	-2.03	4.09
239	NR_015522.1	*2810429I04Rik*	-2.03	4.08
240	NM_001111080.1	*Uhrf1*	-2.03	4.08
241	NM_175347.4	*Srl*	-2.03	4.08
242	NM_030721.2	*Acox3*	-2.03	4.07
243	NM_001167994.1	*Trmt2b*	-2.03	4.07
244	NM_008253.3	*Hmgb3*	-2.02	4.05
245	NM_175511.4	*Fam78a*	-2.01	4.03

246	NM_027534.2	*Kdsr*	-2.01	4.03
247	NM_198642.2	*5031414D18Rik*	-2.01	4.02
248	NM_026003.2	*Smarca2*	-2	4.01
249	NM_134041.3	*4930427A07Rik*	-2	3.99
250	NM_015810.2	*Polg2*	-2	3.99
251	NM_001109993.1	*Tmem141*	-1.99	3.98
252	NM_007634.4	*Ccnf*	-1.99	3.98
253	NM_145564.3	*Fbxo21*	-1.99	3.97
254	NM_027975.2	*Fam83d*	-1.99	3.96
255	NM_001033484.1	*Iqgap3*	-1.99	3.96
256	NM_133669.4	*Rp2h*	-1.98	3.95
257	NM_001113574.1	*Brd3*	-1.98	3.94
258	NM_010580.2	*Itgb5*	-1.98	3.94
259	NM_025998.3	*Nkain1*	-1.98	3.94
260	NM_020271.3	*Pdxp*	-1.98	3.94
261	NM_130895.3	*Adarb1*	-1.98	3.94
262	NM_008567.1	*Mcm6*	-1.98	3.94
263	NM_007705.2	*Cirbp*	-1.98	3.94
264	NM_010559.2	*Il6ra*	-1.98	3.93
265	NM_197943.2	*Sgsm2*	-1.97	3.93
266	NM_001013026.2	*Ttf2*	-1.97	3.92
267	NM_177372.3	*Dna2*	-1.97	3.91
268	NM_023229.2	*Fastk*	-1.96	3.9
269	NM_001199213.1	*Serpinb12*	-1.96	3.89
270	NM_198299.1	*E130303B06Rik*	-1.96	3.88
271	NM_027106.4	*Avpi1*	-1.96	3.88
272	NM_016974.3	*Dbp*	-1.95	3.87
273	NM_008618.3	*Mdh1*	-1.95	3.87
274	NM_010637.3	*Klf4*	-1.95	3.86
275	NM_001199124.1	*Spc25*	-1.95	3.85
276	NM_177583.4	*Aph1b*	-1.94	3.84
277	NM_028657.3	*F630110N24Rik*	-1.94	3.84
278	NM_009104.2	*Rrm2*	-1.94	3.84
279	NM_031182.2	*Tfap4*	-1.93	3.82
280	NM_001177867.1	*Sgol2*	-1.93	3.82
281	NM_013770.2	*Slc25a10*	-1.93	3.82
282	NM_173437.2	*Nav1*	-1.93	3.82

283	NM_178856.1	*Gins2*	-1.93	3.81
284	NM_207204.2	*Ninl*	-1.93	3.81
285	NM_010830.2	*Msh6*	-1.93	3.8
286	NM_025857.2	*Aagab*	-1.93	3.8
287	NM_145509.2	*5430435G22Rik*	-1.92	3.79
288	NM_178214.3	*Hist2h2be*	-1.92	3.79
289	NM_008695.2	*Nid2*	-1.92	3.79
290	NM_008323.1	*Idh3g*	-1.92	3.78
291	NM_009183.2	*St8sia4*	-1.92	3.78
292	NM_001130412.1	*Lpin1*	-1.91	3.77
293	NM_001033311.3	*Vsig10*	-1.91	3.76
294	NM_133345.2	*Ing4*	-1.91	3.75
295	NM_010313.1	*Gnb5*	-1.91	3.75
296	NM_172443.3	*Tbc1d16*	-1.91	3.75
297	NM_029467.3	*Tcam1*	-1.9	3.74
298	NM_011861.2	*Pacsin1*	-1.9	3.74
299	NM_007435.1	*Abcd1*	-1.9	3.73
300	NM_009734.3	*Azi1*	-1.9	3.73
301	NM_007956.4	*Esr1*	-1.89	3.71
302	NM_144915.3	*Daglb*	-1.89	3.71
303	NM_008921.2	*Prim1*	-1.89	3.71
304	NM_011416.2	*Smarca2*	-1.89	3.71
305	NM_022654.1	*Lrdd*	-1.89	3.71
306	NM_021790.1	*Cenpk*	-1.89	3.7
307	NM_025495.3	*Cenpp*	-1.89	3.69
308	NM_025377.3	*Fam33a*	-1.88	3.69
309	NM_001012517.4	*Fut10*	-1.88	3.67
310	NM_172903.4	*Man2a2*	-1.88	3.67
311	NM_001163488.1	*Pfkm*	-1.88	3.67
312	NM_008521.1	*Ltc4s*	-1.87	3.67
313	NM_025512.2	*Zfand1*	-1.87	3.66
314	NM_008845.4	*Pip4k2a*	-1.87	3.66
315	NM_178694.3	*Zer1*	-1.87	3.66
316	NM_011133.2	*Pole2*	-1.87	3.66
317	NM_001199433.1	*Dnmt1*	-1.87	3.66
318	NM_031392.2	*Wdr6*	-1.87	3.65
319	NM_028603.4	*Zbtb8a*	-1.87	3.65

320	NM_029249.2	*4930547N16Rik*	-1.87	3.65
321	NM_027560.1	*Arrdc2*	-1.87	3.65
322	NM_028651.2	*Tmtc4*	-1.87	3.65
323	NM_133249.2	*Ppargc1b*	-1.87	3.65
324	NM_145142.2	*Chst10*	-1.86	3.64
325	NM_001081237.1	*Klhdc5*	-1.86	3.63
326	NM_001039000.4	*Kif5a*	-1.86	3.63
327	NM_145924.2	*Cenpi*	-1.86	3.63
328	NM_030717.1	*Lactb*	-1.86	3.62
329	NM_133765.4	*Fbxo31*	-1.86	3.62
330	NM_026418.2	*Rgs10*	-1.86	3.62
331	NM_145475.4	*Cerk*	-1.85	3.61
332	NM_001010833.2	*Mdc1*	-1.85	3.61
333	NM_178061.5	*Mob3b*	-1.85	3.6
334	NM_177327.3	*Wwp1*	-1.85	3.6
335	NM_001081076.2	*Gucy2g*	-1.85	3.59
336	NM_022882.4	*Lpin2*	-1.84	3.59
337	NM_001142647.1	*Tmem194b*	-1.84	3.58
338	NR_030718.1	*F630028O10Rik*	-1.84	3.58
339	NM_001081433.3	*Ankrd44*	-1.84	3.58
340	NM_020022.2	*Rfc2*	-1.84	3.58
341	NM_010455.2	*Hoxa7*	-1.84	3.58
342	NM_133719.2	*Metrn*	-1.84	3.58
343	NM_145508.2	*Dyrk3*	-1.84	3.58
344	NM_030251.3	*Abtb1*	-1.84	3.57
345	NM_011496.1	*Aurkb*	-1.84	3.57
346	NM_001080926.1	*Lrp8*	-1.83	3.56
347	NM_153781.1	*Pygb*	-1.83	3.56
348	NM_011158.3	*Prkar2b*	-1.83	3.56
349	NM_001081406.1	*Lrr1*	-1.83	3.55
350	NM_145946.2	*Fanci*	-1.83	3.55
351	NM_001039139.1	*Camk2g*	-1.83	3.55
352	NM_172468.2	*Snx30*	-1.83	3.55
353	NM_001164369.1	*Bcas1*	-1.82	3.54
354	NM_026708.1	*Tlcd1*	-1.82	3.54
355	NM_010478.2	*Hspa1b*	-1.82	3.54
356	NM_001081069.1	*Rgs11*	-1.82	3.54

357	NM_024184.2	*Asf1b*	-1.82	3.54
358	NM_001145884.1	*Itgb5*	-1.82	3.53
359	NM_175380.5	*Gpd1l*	-1.82	3.53
360	NM_175445.4	*Rassf2*	-1.82	3.53
361	NM_026086.2	*Nanp*	-1.82	3.53
362	NM_001002267.2	*Tmem158*	-1.82	3.52
363	NM_001163518.1	*2410075B13Rik*	-1.81	3.52
364	NM_018739.2	*Rp9*	-1.81	3.51
365	NM_008568.2	*Mcm7*	-1.81	3.51
366	NM_001004362.2	*2610008E11Rik*	-1.81	3.51
367	NM_029850.3	*Bcl7a*	-1.81	3.5
368	NM_026281.2	*Tm7sf3*	-1.81	3.5
369	NM_001159559.1	*Xrcc6bp1*	-1.8	3.49
370	NM_027995.2	*Paqr7*	-1.8	3.49
371	NM_030246.2	*Dcaf4*	-1.8	3.49
372	NM_001004164.2	*Gnptab*	-1.8	3.49
373	NM_001177804.1	*Sirt3*	-1.8	3.48
374	NM_010615.1	*Kif11*	-1.8	3.48
375	NM_183091.3	*Tonsl*	-1.8	3.48
376	NM_019551.2	*Tdp2*	-1.8	3.48
377	NM_145998.3	*Hmx2*	-1.79	3.47
378	NM_010790.2	*Melk*	-1.79	3.46
379	NM_022419.3	*Abhd8*	-1.79	3.45
380	NM_019488.4	*Slc2a8*	-1.79	3.45
381	NM_025979.4	*Mastl*	-1.79	3.45
382	NM_027667.3	*Arhgap19*	-1.79	3.45
383	NM_170755.2	*Fam134a*	-1.79	3.45
384	NM_134054.2	*1110002B05Rik*	-1.79	3.45
385	NM_001025311.1	*St6galnac6*	-1.78	3.45
386	NM_013692.2	*Klf10*	-1.78	3.44
387	NM_001081266.1	*Ccdc142*	-1.78	3.44
388	NM_019482.2	*Panx1*	-1.78	3.44
389	NM_001146081.1	*Fancb*	-1.78	3.43
390	NM_145392.2	*Bag2*	-1.78	3.43
391	NM_178113.3	*Ncapd3*	-1.78	3.43
392	NR_028479.1	*Snora20*	-1.78	3.43
393	NM_001082974.2	*Neurl2*	-1.78	3.42

394	NM_145853.2	*Tpcn1*	-1.77	3.41
395	NM_001043322.1	*Fmn1*	-1.77	3.41
396	NM_001177867.1	*Sgol2*	-1.77	3.41
397	NM_026376.3	*Plxnd1*	-1.77	3.41
398	NM_026975.2	*Bola1*	-1.77	3.41
399	NM_134081.5	*Dnajc9*	-1.77	3.41
400	NM_025666.2	*Ubr7*	-1.77	3.41
401	NM_172513.3	*Fam126b*	-1.77	3.41
402	NM_011340.3	*Serpinf1*	-1.77	3.4
403	NM_011284.3	*Rpa2*	-1.77	3.4
404	NM_008057.3	*Fzd7*	-1.77	3.4
405	NM_027480.3	*Ankrd24*	-1.76	3.4
406	NM_001025371.2	*Serinc4*	-1.76	3.39
407	NM_139303.1	*Kif18a*	-1.76	3.39
408	NM_011945.2	*Map3k1*	-1.76	3.39
409	NM_019972.2	*Sort1*	-1.76	3.39
410	NM_011132.2	*Pole*	-1.76	3.39
411	NM_008679.3	*Ncoa3*	-1.76	3.39
412	NM_027187.3	*Rnaseh2a*	-1.76	3.38
413	NM_027435.2	*Atad2*	-1.76	3.38
414	NM_009643.1	*Ahnak*	-1.76	3.38
415	NM_001077695.1	*Ncoa2*	-1.76	3.38
416	NM_019484.4	*Alyref2*	-1.76	3.38
417	NM_010353.2	*Gsg2*	-1.76	3.38
418	NM_001164355.1	*Ska1*	-1.76	3.38
419	NM_013538.5	*Cdca3*	-1.76	3.38
420	NM_001164205.1	*Tsen34*	-1.75	3.37
421	NM_001039507.1	*Lipe*	-1.75	3.37
422	NM_001037736.1	*Arhgef10*	-1.75	3.37
423	NM_009516.3	*Wee1*	-1.75	3.37
424	NM_001081066.1	*Dennd3*	-1.75	3.37
425	NM_027986.3	*Cdadc1*	-1.75	3.36
426	NM_001146689.1	*Ezh2*	-1.75	3.36
427	NM_001037725.2	*Fam117b*	-1.75	3.36
428	NM_013907.2	*Fbxw4*	-1.75	3.35
429	NM_178309.2	*Brip1*	-1.74	3.35
430	NM_001128171.1	*Cyld*	-1.74	3.34

431	NM_148958.2	*Osbpl10*	-1.74	3.34
432	NM_023480.2	*Fahd1*	-1.74	3.34
433	NR_027845.1	*Pex11b*	-1.74	3.33
434	NM_001134494.1	*Fam55c*	-1.74	3.33
435	NM_181395.2	*Pxdn*	-1.73	3.33
436	NM_001012401.2	*Hspb6*	-1.73	3.32
437	NM_080793.5	*Setd7*	-1.73	3.32
438	NM_173396.2	*Tgif2*	-1.73	3.31
439	NM_001162855.1	*Nsmce4a*	-1.73	3.31
440	NM_028815.4	*Cep97*	-1.73	3.31
441	NM_016777.3	*Nasp*	-1.73	3.31
442	NM_015733.4	*Casp9*	-1.73	3.31
443	NM_021398.3	*Slc43a3*	-1.72	3.3
444	NM_001081099.1	*2610002D18Rik*	-1.72	3.3
445	NM_172434.2	*Celf3*	-1.72	3.3
446	NM_001110265.1	*Ttk*	-1.72	3.3
447	NM_144852.3	*Slc7a4*	-1.72	3.3
448	NM_145220.2	*Appl2*	-1.72	3.3
449	NM_001039534.1	*Pstk*	-1.72	3.3
450	NM_001199310.1	*Lig1*	-1.72	3.3
451	NM_026260.2	*Tctn3*	-1.72	3.3
452	NM_133971.2	*Ankrd10*	-1.72	3.3
453	NM_019972.2	*Sort1*	-1.72	3.29
454	NM_029096.3	*1110008J03Rik*	-1.72	3.29
455	NM_025866.3	*Cdca7*	-1.72	3.29
456	NM_001163608.1	*Plxdc1*	-1.72	3.28
457	NM_001025365.2	*Miip*	-1.72	3.28
458	NM_053273.2	*Ttyh2*	-1.71	3.28
459	NM_001159646.1	*Dut*	-1.71	3.28
460	NM_025676.3	*Mcm8*	-1.71	3.28
461	NM_001122958.1	*Rad54l*	-1.71	3.28
462	NM_001039390.2	*Pkig*	-1.71	3.28
463	NM_177583.4	*Aph1b*	-1.71	3.27
464	NM_009445.2	*Ttk*	-1.71	3.27
465	NM_145076.3	*Trim24*	-1.71	3.26
466	NM_177167.4	*Ppm1e*	-1.71	3.26
467	NM_153820.3	*Arhgap15*	-1.71	3.26

468	NM_001177985.1	*Zmym3*	-1.71	3.26
469	NM_001114085.1	*Nde1*	-1.7	3.26
470	NM_058214.3	*Recql4*	-1.7	3.26
471	NM_028131.3	*Cenpn*	-1.7	3.26
472	NM_144547.2	*Amhr2*	-1.7	3.26
473	NM_028056.1	*1600002H07Rik*	-1.7	3.25
474	NM_029249.2	*4930547N16Rik*	-1.7	3.25
475	NM_010892.3	*Nek2*	-1.7	3.25
476	NM_012027.2	*Mprip*	-1.7	3.25
477	NM_024248.1	*Cars2*	-1.7	3.24
478	NM_009151.3	*Selplg*	-1.7	3.24
479	NM_031397.2	*Bicc1*	-1.7	3.24
480	NM_001163359.1	*Fignl1*	-1.7	3.24
481	NM_013872.3	*Pmm1*	-1.69	3.24
482	NM_173437.2	*Nav1*	-1.69	3.23
483	NM_145409.2	*Chtf18*	-1.69	3.22
484	NM_001033244.3	*Fancd2*	-1.69	3.22
485	NM_007610.1	*Casp2*	-1.68	3.21
486	NM_172578.2	*Mis18bp1*	-1.68	3.21
487	NM_198423.3	*Bahcc1*	-1.68	3.21
488	NM_027269.3	*1110034A24Rik*	-1.68	3.21
489	NM_001115130.1	*Zbtb44*	-1.68	3.2
490	NM_008535.2	*Lyl1*	-1.68	3.2
491	NM_011932.2	*Dapp1*	-1.68	3.2
492	NM_133926.2	*Camk1*	-1.68	3.2
493	NR_028081.1	*1700040I03Rik*	-1.68	3.2
494	NM_134471.4	*Kif2c*	-1.68	3.19
495	NM_001081170.1	*Pacs2*	-1.67	3.19
496	NM_021534.3	*Pxmp4*	-1.67	3.19
497	NM_001081373.2	*Cep164*	-1.67	3.18
498	NM_011206.2	*Ptpn18*	-1.67	3.18
499	NM_030750.3	*Sgpp1*	-1.67	3.18
500	NM_145588.1	*Kif22*	-1.67	3.18
501	NM_011497.3	*Aurka*	-1.67	3.18
502	NM_022980.4	*Rcan3*	-1.67	3.18
503	NM_001114328.1	*Ccpg1*	-1.67	3.17
504	NM_001130163.1	*Oxr1*	-1.66	3.17

505	NM_027271.1	*D3Ertd751e*	-1.66	3.17
506	NM_001040695.1	*Uevld*	-1.66	3.16
507	NM_018747.4	*Akap7*	-1.66	3.16
508	NM_030026.2	*Mccc2*	-1.66	3.16
509	NM_175027.4	*Fancb*	-1.66	3.16
510	NM_010286.3	*Tsc22d3*	-1.66	3.15
511	NM_027495.4	*Tmem144*	-1.65	3.15
512	NM_146171.1	*Ncapd2*	-1.65	3.15
513	NM_001113364.1	*Tbc1d14*	-1.65	3.15
514	NM_026282.5	*Spc24*	-1.65	3.14
515	NM_001081117.2	*Mki67*	-1.65	3.13
516	NM_025560.2	*1810049H13Rik*	-1.65	3.13
517	NM_001206684.1	*2610528A11Rik*	-1.64	3.13
518	NM_001042653.1	*Oip5*	-1.64	3.12
519	NM_007678.3	*Cebpa*	-1.64	3.12
520	NM_025508.4	*Gmpr*	-1.64	3.11
521	NM_199197.1	*Rbfa*	-1.64	3.11
522	NM_026431.2	*1810043G02Rik*	-1.64	3.11
523	NM_013733.3	*Chaf1a*	-1.64	3.11
524	NM_009547.2	*Zfp161*	-1.64	3.11
525	NM_178683.4	*Depdc1b*	-1.64	3.11
526	NM_001109045.1	*Aqp8*	-1.63	3.1
527	NM_029835.1	*5730590G19Rik*	-1.63	3.1
528	NM_175275.3	*Cntln*	-1.63	3.1
529	NM_177026.1	*Tmcc3*	-1.63	3.1
530	NM_001145898.1	*BC052040*	-1.63	3.1
531	NM_026024.2	*Ube2t*	-1.63	3.1
532	NM_001142938.1	*AK010878*	-1.63	3.09
533	NM_011234.4	*Rad51*	-1.63	3.09
534	NM_011605.2	*Tmpo*	-1.63	3.09
535	NM_133765.4	*Fbxo31*	-1.63	3.09
536	NM_021428.4	*Dexi*	-1.62	3.08
537	NM_175152.4	*Thap3*	-1.62	3.08
538	NM_008841.2	*Pik3r2*	-1.62	3.07
539	NM_174987.4	*1810063B05Rik*	-1.62	3.07
540	NM_001167967.1	*Ankrd10*	-1.62	3.07
541	NM_001162415.1	*Pfkfb2*	-1.62	3.07

542	NM_033146.1	*Fam158a*	-1.62	3.07
543	NM_183046.1	*Kif20b*	-1.62	3.07
544	NM_144818.3	*Ncaph*	-1.61	3.06
545	NM_027258.1	*Rnf157*	-1.61	3.06
546	NM_017376.3	*Tef*	-1.61	3.06
547	NM_008892.2	*Pola1*	-1.61	3.05
548	NR_037773.1	*Tmem41a*	-1.61	3.05
549	NM_008793.2	*Pcsk4*	-1.61	3.05
550	NM_183220.2	*Accs*	-1.61	3.05
551	NM_008063.2	*Slc37a4*	-1.61	3.05
552	NM_009399.3	*Tnfrsf11a*	-1.61	3.05
553	NM_009352.2	*Terf1*	-1.61	3.05
554	NM_028128.1	*Rfc5*	-1.6	3.04
555	NM_001163502.1	*C130039O16Rik*	-1.6	3.04
556	NM_172543.4	*Fam117a*	-1.6	3.03
557	NM_028119.5	*Ddb2*	-1.6	3.03
558	NM_001163794.1	*Pdik1l*	-1.6	3.03
559	NM_001009951.1	*BC088983*	-1.6	3.03
560	NM_007398.3	*Ada*	-1.6	3.03
561	NM_001098528.2	*Kcnb2*	-1.6	3.02
562	NM_010347.3	*Aes*	-1.59	3.02
563	NM_010150.2	*Nr2f6*	-1.59	3.02
564	NM_199308.2	*Mast3*	-1.59	3.02
565	NM_028481.1	*Ccdc18*	-1.59	3.02
566	NR_002847.2	*Malat1*	-1.59	3.01
567	NM_020046.3	*Dhodh*	-1.59	3.01
568	NM_001130526.1	*Lzts2*	-1.59	3.01
569	NM_013769.2	*Tjp3*	-1.59	3.01
570	NM_001127382.1	*Rbm47*	-1.59	3.01
571	NM_025964.3	*Mettl21a*	-1.59	3.01
572	NM_001014976.2	*Espl1*	-1.59	3.01
573	NM_001164366.1	*Atp13a2*	-1.59	3
574	NM_030719.3	*Gatsl2*	-1.59	3
575	NM_023317.2	*Nde1*	-1.59	3
576	NM_028101.4	*Jmjd8*	-1.59	3
577	NM_001081453.1	*Nin*	-1.59	3
578	NM_025411.4	*1110049F12Rik*	-1.58	3

579	NM_009013.3	*Rad51ap1*	-1.58	3
580	NM_028006.2	*Tube1*	-1.58	2.99
581	NM_030708.2	*Zfhx4*	-1.58	2.99
582	NM_001146073.1	*Hexdc*	-1.58	2.99
583	NM_009863.2	*Cdc7*	-1.58	2.99
584	NM_011131.3	*Pold1*	-1.58	2.99
585	NM_133835.2	*Ubac1*	-1.58	2.99
586	NM_080446.2	*Helb*	-1.58	2.99
587	NM_001145780.1	*Use1*	-1.58	2.98
588	NM_201367.3	*Gpr176*	-1.58	2.98
589	NM_025853.3	*Dsn1*	-1.57	2.97
590	NM_198654.3	*Nsl1*	-1.57	2.97
591	NM_001146180.1	*Mtss1*	-1.57	2.97
592	NM_013829.2	*Plcb4*	-1.57	2.97
593	NM_027715.1	*Otud1*	-1.57	2.97
594	NM_145573.2	*Mrps35*	-1.57	2.97
595	NM_001164112.1	*Nfatc1*	-1.57	2.97
596	NM_019479.3	*Hes6*	-1.57	2.96
597	NM_146033.3	*Ankmy2*	-1.57	2.96
598	NM_174876.3	*Impg2*	-1.57	2.96
599	NM_009644.2	*Ahrr*	-1.57	2.96
600	NM_001159581.1	*Mutyh*	-1.56	2.96
601	XR_105811.1	*A230045G11Rik*	-1.56	2.96
602	NM_011919.4	*Ing1*	-1.56	2.95
603	NM_029988.2	*Pigh*	-1.56	2.95
604	NM_133215.1	*Mtmr4*	-1.56	2.95
605	NM_001033175.2	*Cln6*	-1.56	2.95
606	NM_144804.1	*Depdc7*	-1.56	2.95
607	NM_001033273.2	*5031439G07Rik*	-1.56	2.95
608	NM_008026.4	*Fli1*	-1.56	2.95
609	NM_024245.4	*Kif23*	-1.56	2.95
610	NM_008566.2	*Mcm5*	-1.56	2.95
611	NM_011249.2	*Rbl1*	-1.56	2.94
612	NM_001160145.1	*Tmem9*	-1.56	2.94
613	NM_177466.4	*Rab11fip5*	-1.55	2.93
614	NM_011121.3	*Plk1*	-1.55	2.93
615	NM_001163042.1	*Haus8*	-1.55	2.93

616	NM_007499.2	*Atm*	-1.55	2.92
617	NM_011397.4	*Slc23a1*	-1.55	2.92
618	NM_177460.4	*Parp16*	-1.55	2.92
619	NM_026321.4	*Fam174a*	-1.55	2.92
620	NM_021506.2	*Sh3rf1*	-1.54	2.91
621	NM_145928.1	*Tspan14*	-1.54	2.91
622	NM_001081001.1	*Brca2*	-1.54	2.91
623	NM_001040686.1	*Zfp692*	-1.54	2.9
624	NM_016785.2	*Tpmt*	-1.54	2.9
625	NR_015572.1	*1810014B01Rik*	-1.53	2.9
626	NM_030087.2	*Ndufv3*	-1.53	2.89
627	NM_178732.3	*Zfp324*	-1.53	2.89
628	NM_001039556.3	*Rad54b*	-1.53	2.89
629	NM_001163476.1	*Gins1*	-1.53	2.88
630	NM_001081117.2	*Mki67*	-1.53	2.88
631	NM_026129.2	*Erp29*	-1.53	2.88
632	NM_019990.4	*Stard10*	-1.52	2.88
633	NM_080850.2	*Pask*	-1.52	2.87
634	NM_138583.2	*D16H22S680E*	-1.52	2.87
635	NM_001178013.1	*Sfxn3*	-1.52	2.87
636	NM_001161737.1	*Siva1*	-1.52	2.87
637	NM_134042.2	*Aldh6a1*	-1.52	2.87
638	NM_177171.4	*Heatr5a*	-1.52	2.87
639	NM_023294.2	*Ndc80*	-1.52	2.87
640	NM_027009.2	*Rfc3*	-1.52	2.86
641	NM_001204273.1	*Lsm2*	-1.52	2.86
642	NM_001012273.1	*Birc5*	-1.52	2.86
643	NM_009569.3	*Zfpm1*	-1.51	2.86
644	NM_183321.1	*BC053749*	-1.51	2.85
645	NM_146256.3	*Hpdl*	-1.51	2.85
646	NM_053269.3	*Rad51c*	-1.51	2.85
647	NM_178911.4	*Pld4*	-1.51	2.85
648	NM_001163422.1	*Tatdn3*	-1.51	2.85
649	NM_026512.1	*Bphl*	-1.51	2.85
650	NM_001039959.1	*Ahnak*	-1.51	2.85
651	NM_198105.2	*Fam120c*	-1.51	2.84
652	NM_019798.5	*Pde4a*	-1.51	2.84

653	NM_176979.5	*Topbp1*	-1.51	2.84
654	NM_028875.2	*Xrcc3*	-1.5	2.84
655	NM_011623.2	*Top2a*	-1.5	2.83
656	NM_028634.3	*Cby1*	-1.5	2.83
657	NM_013753.2	*X99384*	-1.5	2.83
658	NM_019940.2	*Zfp111*	-1.5	2.83
659	NM_026282.5	*Spc24*	-1.5	2.83
660	NM_001012273.1	*Birc5*	-1.5	2.83
661	NM_146067.3	*Cpped1*	-1.5	2.83
662	NM_177752.4	*Eme1*	-1.5	2.82
663	NM_009689.2	*Birc5*	-1.49	2.82
664	NM_001081099.1	*2610002D18Rik*	-1.49	2.82
665	NM_001113470.1	*Ctdsp2*	-1.49	2.81
666	NM_011906.2	*Tpra1*	-1.49	2.81
667	NM_007533.5	*Bckdha*	-1.49	2.81
668	NM_008044.2	*Fxn*	-1.49	2.8
669	NM_181815.3	*4930534B04Rik*	-1.48	2.8
670	NM_177343.3	*Camk1d*	-1.48	2.8
671	NM_008637.1	*Nudt1*	-1.48	2.8
672	NM_001039669.2	*Iffo1*	-1.48	2.8
673	NM_011734.3	*Siae*	-1.48	2.8
674	NM_001253809.1	*Racgap1*	-1.48	2.79
675	NM_026543.3	*3010026O09Rik*	-1.48	2.79
676	NM_025812.2	*Hmg20a*	-1.48	2.79
677	NM_212473.1	*Fam53b*	-1.48	2.79
678	NM_175265.4	*6720463M24Rik*	-1.48	2.79
679	NM_010829.2	*Msh3*	-1.48	2.79
680	NM_001081499.2	*Tbc1d8b*	-1.48	2.78
681	NM_011871.2	*Prkra*	-1.48	2.78
682	NM_008565.3	*Mcm4*	-1.47	2.77
683	NM_001195486.1	*Srsf7*	-1.47	2.77
684	NM_001039562.1	*Ankrd37*	-1.47	2.77
685	NM_027855.3	*0610007C21Rik*	-1.47	2.77
686	NM_011894.2	*Sh3bp5*	-1.47	2.77
687	NM_028536.1	*1700054N08Rik*	-1.47	2.77
688	NM_001081396.2	*Wdr67*	-1.47	2.77
689	NM_146089.2	*Haus1*	-1.47	2.77

690	NM_001114085.1	*Nde1*	-1.47	2.76
691	NM_011919.4	*Ing1*	-1.47	2.76
692	NM_080456.1	*Mrps6*	-1.46	2.76
693	NM_133190.1	*Cacng8*	-1.46	2.76
694	NM_178396.4	*Car12*	-1.46	2.75
695	NM_199308.2	*Mast3*	-1.46	2.75
696	NM_001109993.1	*Tmem141*	-1.46	2.75
697	NM_176836.3	*Fam76b*	-1.46	2.75
698	NM_001136073.1	*Nfatc2*	-1.46	2.75
699	NM_024240.6	*Gins4*	-1.46	2.75
700	NM_019800.4	*Acp6*	-1.46	2.74
701	NM_011020.3	*Hspa4l*	-1.45	2.74
702	NM_172824.3	*Ccdc14*	-1.45	2.73
703	NM_153545.2	*Lrrc45*	-1.45	2.73
704	NM_008787.3	*Pcnt*	-1.45	2.73
705	NM_020270.2	*Scamp5*	-1.45	2.73
706	NM_029394.3	*Snx24*	-1.45	2.73
707	NM_001112711.1	*Grk6*	-1.45	2.73
708	NM_016769.4	*Smad3*	-1.45	2.73
709	NM_001081170.1	*Pacs2*	-1.45	2.73
710	NM_009014.3	Rad51l1	-1.45	2.73
711	NM_027745.1	Ccdc57	-1.45	2.72

Table S4. The differentially expressed genes between shDab2-B and shLuc cells (DC/LC)

No.	GenBank accession no.	Gene Symbol	Log_2 Ratio	Fold Change
**Up-regulated genes**				
1	NM_022886.2	*Scel*	2.86	7.24
2	NM_080853.3	*Slc17a6*	2.81	7.00
3	NM_001081076.2	*Gucy2g*	2.69	6.45
4	NM_010215.3	*Il4i1*	2.37	5.16
5	NM_001081180.1	*Spink5*	2.36	5.15
6	NM_011333.3	*Ccl2*	2.20	4.59
7	NM_010658.3	*Mafb*	2.18	4.52
8	NM_020008.2	*Clec7a*	2.17	4.50
9	NM_023061.2	*Mcam*	2.14	4.42
10	NM_174876.3	*Impg2*	2.13	4.37
11	NM_010118.3	*Egr2*	1.99	3.98
12	NM_001146119.1	*Fam49a*	1.96	3.89
13	NM_001199105.1	*Trp53inp1*	1.95	3.86
14	NM_013750.2	*Phlda3*	1.94	3.84
15	NM_021334.2	*Itgax*	1.94	3.83
16	NM_026439.2	*Ccdc80*	1.90	3.74
17	NR_030687.1	*Rn7sk*	1.89	3.72
18	NM_153408.2	*Neurl3*	1.87	3.66
19	NM_008808.3	*Pdgfa*	1.87	3.65
20	NM_011607.3	*Tnc*	1.84	3.59
21	NM_013454.3	*Abca1*	1.82	3.54
22	NM_009344.3	*Phlda1*	1.72	3.29
23	NM_019568.2	*Cxcl14*	1.69	3.22
24	NM_011459.4	*Serpinb8*	1.67	3.19
25	NM_008392.1	*Irg1*	1.60	3.03
26	NM_176913.3	*Dpep2*	1.59	3.01
27	NM_001172055.1	*Bdh2*	1.59	3.00
28	NM_009344.3	*Phlda1*	1.58	2.98
29	NM_017480.2	*Icos*	1.57	2.97
30	NM_009192.2	*Sla*	1.55	2.93

31	NM_008139.5	*Gnaq*	1.52	2.87
32	NM_010130.4	*Emr1*	1.52	2.86
33	NM_001190950.1	*Kcne3*	1.48	2.79
34	NM_011338.2	*Ccl9*	1.47	2.78
**Down-regulated genes**				
1	NM_153543.2	*Aldh1l2*	-2.58	5.99
2	NM_008132.2	*Glrp1*	-2.47	5.55
3	NM_018861.3	*Slc1a4*	-2.43	5.41
4	NM_175093.2	*Trib3*	-2.43	5.38
5	NM_145953.2	*Cth*	-2.32	4.98
6	NM_026929.4	*Chac1*	-2.22	4.66
7	NM_001130479.1	*Nucb2*	-1.92	3.77
8	NM_027258.1	*Rnf157*	-1.86	3.64
9	NM_008483.3	*Lamb2*	-1.71	3.26
10	NM_027641.2	*Spef1*	-1.69	3.23
11	NM_019765.4	*Clip1*	-1.59	3.01
12	NM_008695.2	*Nid2*	-1.56	2.96
13	NM_177981.2	*Hap1*	-1.55	2.94
14	NM_001159538.1	*Fgd2*	-1.55	2.93
15	NM_001130479.1	*Nucb2*	-1.51	2.86
16	NM_008810.2	*Pdha1*	-1.51	2.86
17	NM_001105561.1	*Gm11545*	-1.50	2.82
18	NM_008737.2	*Nrp1*	-1.45	2.73

Table S5. The differentially expressed genes between LPS-treated shDab2-B and shLuc cells (DL/LL)

No.	GenBank accession no.	Gene Symbol	Log_2 Ratio	Fold Change
**Up-regulated genes**				
1	NR_033554.1	*D330041H03Rik*	6.64	100.00
2	NM_031168.1	*Il6*	4.61	24.47
3	NM_001174170.1	*Serpinb2*	3.95	15.43
4	NM_010259.2	*Gbp1*	3.77	13.63
5	NM_008491.1	*Lcn2*	3.74	13.35
6	NM_010260.1	*Gbp2*	3.42	10.70
7	NM_001044384.1	*Timp1*	2.99	7.94
8	NM_020001.2	*Clec4n*	2.80	6.98
9	NM_008485.3	*Lamc2*	2.79	6.93
10	NM_203320.2	*Cxcl3*	2.70	6.51
11	NM_177371.3	*Tnfsf15*	2.64	6.25
12	NM_017466.4	*Ccrl2*	2.63	6.19
13	NM_010846.1	*Mx1*	2.60	6.07
14	NM_001081984.1	*Popdc2*	2.56	5.92
15	NM_009137.2	*Ccl22*	2.56	5.92
16	NM_001159558.1	*Cd36*	2.54	5.81
17	NM_021334.2	*Itgax*	2.53	5.77
18	NM_011331.2	*Ccl12*	2.52	5.74
19	NM_001081180.1	*Spink5*	2.51	5.70
20	NM_001040699.1	*Mtmr7*	2.49	5.64
21	NM_008329.2	*Ifi204*	2.47	5.53
22	NM_001142706.1	*Cfb*	2.41	5.30
23	NM_010554.4	*Il1a*	2.35	5.11
24	NM_010171.3	*F3*	2.34	5.07
25	NM_010510.1	*Ifnb1*	2.33	5.01
26	NM_013454.3	*Abca1*	2.30	4.93
27	NM_172603.3	*Phf11*	2.27	4.83
28	NM_010130.4	*Emr1*	2.27	4.81
29	NM_019450.3	*Il1f6*	2.26	4.80

30	NM_001083312.1	*Gbp7*	2.26	4.79
31	NM_001005858.3	*I830012O16Rik*	2.24	4.73
32	NM_020008.2	*Clec7a*	2.23	4.68
33	NM_018734.3	*Gbp3*	2.22	4.67
34	NM_001166376.1	*Ms4a6c*	2.17	4.50
35	NM_009373.3	*Tgm2*	2.16	4.47
36	NM_021274.2	*Cxcl10*	2.13	4.38
37	NM_013654.3	*Ccl7*	2.13	4.38
38	NM_010104.3	*Edn1*	2.13	4.37
39	NM_153510.3	*Pilra*	2.12	4.35
40	NM_019448.3	*Dnmt3l*	2.11	4.32
41	NM_019568.2	*Cxcl14*	2.10	4.28
42	NM_008230.5	*Hdc*	2.09	4.27
43	NM_080853.3	*Slc17a6*	2.08	4.21
44	NM_028595.4	*Ms4a6c*	2.04	4.13
45	NM_009344.3	*Phlda1*	2.04	4.12
46	NM_008331.3	*Ifit1*	2.00	4.00
47	NM_001037917.2	*Gm6377*	1.91	3.77
48	NM_175026.3	*Pyhin1*	1.87	3.66
49	NM_001159402.1	*Upp1*	1.86	3.62
50	NM_009895.3	*Cish*	1.84	3.57
51	NM_011333.3	*Ccl2*	1.83	3.54
52	NM_007498.3	*Atf3*	1.82	3.54
53	NM_030565.6	*Fam20c*	1.81	3.50
54	NM_009969.4	*Csf2*	1.79	3.47
55	NM_009627.1	*Adm*	1.79	3.46
56	NM_001013761.1	*Gm606*	1.79	3.45
57	NM_010329.2	*Pdpn*	1.78	3.44
58	NM_001002927.2	*Penk*	1.78	3.43
59	NM_009192.2	*Sla*	1.77	3.42
60	NM_011607.3	*Tnc*	1.77	3.41
61	NM_011610.3	*Tnfrsf1b*	1.77	3.41
62	NM_177371.3	*Tnfsf15*	1.77	3.41
63	NM_010442.2	*Hmox1*	1.75	3.37
64	NM_176913.3	*Dpep2*	1.74	3.34
65	NM_008215.2	*Has1*	1.74	3.33

66	NM_172803.2	*Dock4*	1.73	3.33
67	NM_008964.4	*Ptger2*	1.72	3.29
68	NM_009856.2	*Cd83*	1.69	3.23
69	NM_028096.1	*2010300C02Rik*	1.69	3.23
70	NM_021394.2	*Zbp1*	1.67	3.18
71	NM_008392.1	*Irg1*	1.66	3.15
72	NM_145066.4	*Gpr85*	1.65	3.14
73	NM_019466.3	*Rcan1*	1.63	3.10
74	NM_153564.2	*Gbp5*	1.63	3.09
75	NR_027805.1	*A430093F15Rik*	1.62	3.07
76	NM_009855.2	*Cd80*	1.61	3.05
77	NM_026835.2	*Ms4a6d*	1.61	3.05
78	NM_013673.3	*Sp100*	1.60	3.03
79	NM_011610.3	*Tnfrsf1b*	1.59	3.02
80	NM_010821.1	*Mpeg1*	1.59	3.01
81	NM_023120.4	*Gnb1l*	1.58	2.98
82	NM_001128133.1	*Cd200r3*	1.57	2.96
83	NM_010215.3	*Il4i1*	1.56	2.94
84	NM_010720.3	*Lipg*	1.55	2.94
85	NM_009705.3	*Arg2*	1.55	2.93
86	NM_025992.2	*Herc6*	1.55	2.93
87	XM_003086779.1	*Gm11428*	1.55	2.92
88	NM_133914.2	*Rasa4*	1.54	2.92
89	NM_008139.5	*Gnaq*	1.54	2.91
90	NM_007836.1	*Gadd45a*	1.54	2.90
91	NM_001146119.1	*Fam49a*	1.53	2.89
92	NM_021384.4	*Rsad2*	1.52	2.87
93	NM_001113527.1	*Isg20*	1.52	2.86
94	NM_025586.3	*Rpl15*	1.51	2.84
95	NM_008538.2	*Marcks*	1.51	2.84
96	NM_207231.1	*Arl5c*	1.49	2.82
97	NM_020260.2	*Arhgap31*	1.49	2.81
98	NM_134102.4	*Pla1a*	1.48	2.78
99	NM_001081076.2	*Gucy2g*	1.47	2.77
100	NM_011299.4	*Rps6ka2*	1.47	2.76
101	NM_025626.4	*Fam107b*	1.46	2.76

102	NM_008321.2	*Id3*	1.46	2.75
103	NM_013653.3	*Ccl5*	1.45	2.74
104	NM_008332.3	*Ifit2*	1.45	2.74
**Down-regulated genes**				
1	NM_001105561.1	*Gm11545*	-2.41	5.32
2	XR_140864.1	*5330417H12Rik*	-2.30	4.92
3	NM_134050.4	*Rab15*	-2.07	4.21
4	NM_001164717.1	*Sh3pxd2a*	-1.97	3.93
5	NM_177235.3	*Bend6*	-1.95	3.88
6	NM_181039.2	*Lphn1*	-1.92	3.79
7	NM_029116.2	*Kbtbd11*	-1.84	3.59
8	NM_027118.1	*Cdk13*	-1.78	3.44
9	NM_178396.4	*Car12*	-1.76	3.39
10	NM_177778.4	*Armc7*	-1.74	3.33
11	NM_008695.2	*Nid2*	-1.69	3.22
12	NM_178856.1	*Gins2*	-1.68	3.20
13	NM_016803.3	*Chst3*	-1.65	3.13
14	NM_019429.2	*Prss16*	-1.64	3.13
15	NM_146094.2	*Fads1*	-1.64	3.11
16	NM_007421.2	*Adssl1*	-1.64	3.11
17	NM_001159538.1	*Fgd2*	-1.59	3.01
18	NM_001033162.2	*1700012A16Rik*	-1.59	3.00
19	NM_181316.4	*Bbs9*	-1.58	3.00
20	NM_008323.1	*Idh3g*	-1.57	2.97
21	NM_008810.2	*Pdha1*	-1.56	2.96
22	NM_001145821.1	*Ggta1*	-1.56	2.95
23	NM_148958.2	*Osbpl10*	-1.53	2.89
24	NM_001080926.1	*Lrp8*	-1.53	2.89
25	NM_015733.4	*Casp9*	-1.53	2.88
26	NM_177167.4	*Ppm1e*	-1.52	2.86
27	NM_001033171.2	*Klrg2*	-1.50	2.82
28	NM_011110.4	*Pla2g5*	-1.48	2.80
29	NM_145953.2	*Cth*	-1.47	2.76
30	NM_001143777.1	*Fam13c*	-1.46	2.76
31	NM_001130479.1	*Nucb2*	-1.46	2.76
32	NM_025995.2	*Fbxo5*	-1.46	2.75
